# Assessing Inhibitory Control Deficits in Adult ADHD: A Systematic Review and Meta-analysis of the Stop-signal Task

**DOI:** 10.1007/s11065-023-09592-5

**Published:** 2023-06-10

**Authors:** Daniel Senkowski, Theresa Ziegler, Mervyn Singh, Andreas Heinz, Jason He, Tim Silk, Robert C. Lorenz

**Affiliations:** 1 Department of Psychiatry and Neurosciences, Charité - Universitätsmedizin Berlin, corporate member of Freie Universität Berlin and Humboldt Universität Zu Berlin, Charité Campus Mitte (CCM), Charitéplatz 1, 10117 Berlin, Germany; 2 Centre for Social and Early Emotional Development (SEED) and School of Psychology, Deakin University, Geelong, Victoria Australia; 3 King’s College London, London, WC2R 2LS UK; 4 Lise Meitner Group for Environmental Neuroscience, Max Planck Institute for Human Development, Lentzeallee 94, 14195 Berlin, Germany

**Keywords:** Attention deficit hyperactivity disorder, Impulsivity, Cognition, Attention, Cognitive deficits, Neuropsychology

## Abstract

In recent years, there has been an increasing quest in improving our understanding of the neurocognitive deficits underlying adult attention-deficit/hyperactivity disorder (ADHD). Current statistical manuals of psychiatric disorders emphasize inattention and hyperactivity-impulsivity symptoms, but empirical studies have also shown consistent alterations in inhibitory control. To date, there is no established neuropsychological test to assess inhibitory control deficits in adult ADHD. A common paradigm for assessing response inhibition is the stop-signal task (SST). Following PRISMA-selection criteria, our systematic review and meta-analysis integrated the findings of 26 publications with 27 studies examining the SST in adult ADHD. The meta-analysis, which included 883 patients with adult ADHD and 916 control participants, revealed reliable inhibitory control deficits, as expressed in prolonged SST response times, with a moderate effect size $g$ = 0.51 (95% CI: 0.376–0.644,$p$ < 0.0001). The deficits were not moderated by study quality, sample characteristics or clinical parameters, suggesting that they may be a phenotype in this disorder. The analyses of secondary outcome measures revealed greater SST omission errors and reduced go accuracy in patients, indicative of altered sustained attention. However, only few (*N* < 10) studies were available for these measures. Our meta-analysis suggests that the SST, in conjunction with other tests and questionnaires, could become a valuable tool for assessing inhibitory control deficits in adult ADHD.

**Supplementary Information:**

The online version contains supplementary material available at 10.1007/s11065-023-09592-5.

## Introduction

Adult attention deficit hyperactivity disorder (ADHD) is a neurodevelopmental condition that emerges during childhood or young adulthood and is characterized by symptoms of inattention and/or hyperactivity-impulsivity (Adler et al., 2017; Asherson et al., 2016). Adults with ADHD have a global prevalence of 2.58% (persistent disorder) and 6.76% (symptomatic disorder) (Song et al., 2021). In clinical practice, adult ADHD is assessed using questionnaires, interviews with relatives and inspection of school certificates. Although neurocognitive deficits are inherent to ADHD, there is still no established test or test battery that is commonly used in the assessment of this disorder (Fried et al., 2021; Nikolas et al., 2019). Nevertheless, neuropsychological tests should be used in patients with presumed cognitive deficits to objectify these deficits during the diagnostic process. To date, there is an emerging quest to establish neurocognitive paradigms as complementary tools in the assessment of adult ADHD.

Early research on ADHD has suggested that deficits in inhibitory control are a primary phenotype in this disorder (Barkley, 1997). Support for the inhibition deficit model has come from studies showing altered executive functions in adult ADHD (Hadas et al., 2021; Linhartová et al., 2021; Nigg et al., 2002; Silverstein et al., 2020). An important aspect of executive functions is inhibitory control, which has often been investigated with the stop-signal task (SST; Verbruggen & Logan, 2008), but also with other paradigms such as the go/no-go task (Fisher et al., 2011). However, there are substantial differences between the two tasks. For instance, the go/no-go task does not include a measure of individual response inhibition speed, which is explicitly obtained in the SST. Moreover, it has been shown that different neural mechanisms underlie stimulus processing in the two tasks (Raud et al., 2020). Hence, the go/no-go task and the SST likely capture different facets of response inhibition and therefore, they should be examined independently. In the current review and meta-analysis, we focused on the SST in adult ADHD (for a meta-analysis on the go/no-go task in combined children, and adult studies, see Wright et al., 2014).

In the SST, participants are instructed to make a forced-choice response following a ‘go-signal’, e.g., an arrow pointing to the left or right, and to respond with a left or right button press, respectively. Crucially, in a small proportion of trials, an auditory or visual ‘stop-signal’ is presented after the go-signal and participants are required to withhold the behavioral response. In most studies an adaptive approach is used to obtain the delay interval between the go-signal and the stop-signal for which the response inhibition rates are around 50% at the individual subject level. Based on this delay interval and the response times to go-trials, the stop-signal reaction time (SSRT) is calculated, which has become an established measure of response inhibition (Logan et al., 2014). A previous meta-analysis of the SSRT, which involved studies in children and adults diagnosed with ADHD, has revealed deficits of moderate effect sizes $\left(g\right)=0.62$ across age groups (Lipszyc & Schachar, 2010). This meta-analysis included 68 studies with children but only 10 studies with adult ADHD. Therefore, the validity of this analysis regarding adult ADHD was limited and the degree of SSRT deficits in adult ADHD remains to be investigated.

Here, we performed a review and meta-analysis that conformed to current PRISMA guidelines focusing on response inhibition deficits, as measured by the SSRT, in adult ADHD. Our analysis included 26 publications with 27 studies, which allowed for a reliable estimation of response inhibition deficits in patients. We performed a quality assessment of the SST following a recent consensus paper (Verbruggen et al., 2019) and estimated the risk of bias (RoB) for each study. We thoroughly examined whether the study quality as well as participant-related and clinical factors influence response inhibition deficits in adult ADHD.

## Methods

The protocol of this systematic review and meta-analysis has been pre-registered in the PROSPERO database (PROSPERO ID: CRD42021266709).

### Study Selection

The following study selection criteria were applied: 1. Patient population: Studies contained at least one group of adult participants (18 +) with a current diagnosis of ADHD in accordance with the DSM criteria (5 or earlier) or Hyperkinetic Disorder according to the ICD (10 or earlier) criteria. Studies investigating populations with only subclinical ADHD symptoms were not included. 2. Control group: Studies must include at least one healthy control group. 3. Experimental task: Response inhibition performance had to be assessed using the SST or the Stop-Change Task, which is a modified version of the SST in which individuals swith to a secondary response after they have inhibited an ongoing response (Verbruggen & Logan, 2009). Studies using atypical SST paradigms such as dual tasks or the selective SST were excluded. Also excluded were studies in which participants received feedback on stop-signal performance, as feedback and reward influence response inhibition (Lipszyc & Schachar, 2010; Slusarek et al., 2001). 4. Outcome measure**:** Sufficient test statistics for the stop-signal reaction time (SSRT) must be provided to calculate standardized mean differences (Hedge’s g). 5. Other criteria: Empirical articles written in English or German language and published or accepted for publication in peer-reviewed journals during the period 2000–2022.

### Search Strategy

To identify relevant articles, an electronic search was conducted up to April 14, 2022 in two major publication databases: Medline and PsycInfo (accessed from EBSCOhost). The following syntax, adapted from Lipszyc and Schachar (2010), was used: [(attention deficit hyperactivity disorder OR ADHD) AND Adult* AND (stop task OR stop signal OR response inhibition OR executive function)]. Limiters were set so that only articles published in peer-reviewed journals in English or German since the 1st of January 2000 were included. Furthermore, reference lists of the identified empirical articles, previous meta-analyses and systematic reviews were scanned to ensure that all relevant articles were captured.

### Study Selection

The study selection process was conducted by two authors (TZ and DS) and included two stages: 1. Initial screening of titles and abstracts using the inclusion and exclusion criteria described above. 2. For the resulting set of studies full texts were obtained and reviewed in detail for eligibility. Screening of eligible articles was performed in Endnote. In case of disagreement between authors regarding the eligibility of studies, studies were screened by other team members and disagreements during the first or the second screening process were discussed until consensus was reached.

### Data Extraction and Outcomes

Data were extracted independently by two authors (TZ and MS). When statistical values were insufficiently reported for the meta-analysis, the authors of the articles were contacted and asked to provide the missing information. The following measures of the SST were extracted from the articles, separately for patients and controls: SSRT as primary outcome; stop commission errors (responding on a stop trial); go discrimination errors (e.g., responding with the left arrow key, even though a rightwards pointing arrow was presented); go omission errors (not responding on a go trial); and go accuracy (percentage of correct go trials) as secondary outcomes. In addition, important variables that might influence behavioral performance in the planned analysis were extracted and tabulated for each study: Age; IQ; percentage of males; ADHD subtype; years of education; comorbidities; medication status; patient recruitment setting.

### Assessments of the SST Validity and Risk of Bias

The validity of the SST and the risk of bias (RoB) in each study are important factors that could influence group differences between patients and controls. Therefore, they were explicitly examined in the current analysis. Both SST validity and RoB were assessed by two independent raters (TZ and MS). In case of disagreement between the authors, a consensus was reached by discussion with other team members. To increase inter-rater reliability, a calibration session was conducted in which the assessments were applied to two articles not included in this review. This allowed the two raters to identify possible sources of disagreement and to decide on rules for the assessment of ambiguous cases (Supplementary Text 1).

Across studies selected for this meta-analysis, there is considerable variability in the administration of the SST, and the analytic procedures used to derive outcome measures. To determine the validity of the SST, we used the recent consensus guide developed by Verbruggen et al. (2019), which provides 12 ‘best practice’ recommendations for the design, implementation and analysis of the SST. We selected four main criteria from this consensus guide and rephrased them into four dichotomous items, i.e., item fulfilled or not, for the critical appraisal (Supplementary Text 2). In case of missing information, the criterion was rated as not fulfilled.

The overall validity of the SST was then rated as follows: < 3 criteria fulfilled = low validity; 3 criteria fulfilled = moderate validity; 4 criteria fulfilled = high validity. Cohen’s unweighted kappa for nominal data were calculated for each item. 

In case of a bias or a prevalence problem, Byrt’s bias and prevalence adjusted kappa were additionally reported (Byrt et al., 1993; Hallgren, 2012). For the RoB assessment, we applied the adapted Hombrados and Waddington criteria that have been recently used for studies with ADHD patients (Hulsbosch et al., 2021): (1) Equivalent group sizes; (2) Use of a diagnostic interview or questionnaires to determine ADHD diagnosis; (3) Sufficient sample sizes; (4) All statistical outcomes are reported; (5) Transparent reporting of the data analysis; (6) Reporting of missing/excluded data. Each item was rated as “good/low RoB”, “satisfactory/moderate RoB”, or “poor/high RoB”. In accordance with the rating system described in the Cochrane Handbook, an overall quality rating of low, moderate or high was assigned to each study based on the following criteria: If at least one of the categories was rated as having a moderate RoB, the overall RoB could only be rated as moderate as well, even if all other categories were rated as having a low RoB. The same principle applied if at least one category was rated as having a high RoB. Cohen’s weighted Kappa was calculated for each individual domain (Cohen, 1968; Hallgren, 2012). After all studies were rated for SST validity and RoB, a overall study quality variable was created combining the RoB ratings and SST validity. Studies with high RoB and low SST validity were rated as having low overall quality, and studies with moderate or low RoB AND moderate or high SST validity were rated as having moderate to high overall quality. Studies characterized by the remaining combinations of RoB and SST validity (low RoB and low SST validity; moderate RoB and low SST validity; high RoB and moderate SST validity; high RoB and high SST validity) were assigned to the category moderate to low overall quality. This categorization was used for subgroup analyses (see below).

### Meta-Analysis

The meta-analysis was carried out in R (version 4.0.3; R Core Team, 2020) and the metafor package (version 3.0.2; Viechtbauer, 2010). Hedges’ $g$ was calculated for each individual study and each outcome (primary outcome SSRT and secondary outcomes) displaying the effect size of the group difference. Given that various sources could account for differences in findings between studies, e.g., examination of different patient samples or use of different SST paradigms, a random-effects model was fitted to the data. Instead of the usual large-sample approximation, the sampling variance was adjusted by taking the sample-size weighted average of the Hedges' $g$ values into the equation, as this approach has been shown to be less biased (Lin & Aloe, 2021). For computing confidence intervals, the method introduced by Knapp and Hartung (2003) was chosen. To assess for heterogeneity, (1) ${\tau }^{2}$ was estimated using the restricted maximum-likelihood estimator (Viechtbauer, 2005), (2) the *Q*-test for heterogeneity and (3) the ${I}^{2}$ statistics (Higgins & Thompson, 2002) are reported. If heterogeneity between studies is present, i.e., ${\widehat{\tau }}^{2}>0$, regardless of whether the *Q*-test reaches significance, a prediction interval for the true outcomes is provided (Riley et al., 2011). The results will be visualized using forest plots. Furthermore, the model is assessed regarding (1) potential outliers, i.e. studies with studentized residuals larger than the $100\times (1-0.05/(2\times k))th$ percentile of a standard normal distribution, considering a Bonferroni correction with $\alpha=0.05$ (two-sided) for k included studies as well as (2) potentially overinfluential studies, i.e. with a Cook’s distance larger than the median plus 6 times the interquartile range of the Cook’s distances (Viechtbauer & Cheung, 2010). If outliers were detected, leave-one-out diagnostics for sensitivity analysis were conducted.

### Assessment of Publication Bias

Evidence of publication bias was assessed using a combination of visual and statistical approaches. First, the funnel plot (Copas & Chi, 2000) of standardized mean difference (SMD) against the inverse square root of the sample size was visually inspected for asymmetries (Zwetsloot et al., 2017). In the absense of bias, the funnel plot should be symmetrical and narrow down at the top, where studies with larger sample sizes are located and the effect estimate is more precise. However, determining of publication bias using visual inspection methods (such as funnel plots) is often subjective and prone to judgment errors (Wang & Bushman, 1998). Therefore, it is recommended to additionally compute a quantile–quantile plot (Q-Q plot) to aid in the assessment of publication bias. Next, Egger’s regression test was computed using the inverse of the square root sample size as a predictor to statistically test for asymmetry of the funnel plot (Zwetsloot et al., 2017).

### Meta-Regression and Subgroup Analysis

To assess whether the pre-specified extracted demographic and clinical variables as well as study quality influenced the meta-analytic outcome and to explore the source of potential heterogeneity, a meta-regression analysis was performed for continuous (age, sex, IQ) and a subgroup analysis for categorical covariates (RoB, SST validity and overall study quality, comorbidities, patient setting and medication status).

Mixed-effects models were fitted to the data for the meta-regression analysis. If sufficient data were available across studies, the extracted variables were included in a multivariate regression model. Otherwise, univariate models were fitted. The parameter ${\tau }^{2}$, which indicates the residual heterogeneity not explained by the included moderators (Viechtbauer, 2010), was estimated using the REML-estimator (Viechtbauer, 2005). Tests and confidence intervals were calculated by the Knapp and Hartung (2003) method. The mean values for age, IQ, and percentage of males of the study samples were computed for inclusion in a regression model. For this purpose, the reported means of the patients and the means of the controls were averaged. When sample sizes of ADHD participants and healthy controls differed substantially, a sample-size weighted mean was calculated. When IQ scores were reported separately for verbal and non-verbal IQ in a study, the mean of these scores was calculated. IQ was centered before taken into a univariate regression model. Age and gender were standardized before taken into a multivariate regression model.

The following variables were considered for subgroup analysis: (1) RoB with the levels low RoB vs. moderate RoB vs. high RoB; (2) SST validity with the levels low validity vs. moderate validity vs. high validity; (3) overall study quality with the levels low overall quality vs. low to moderate overall quality vs. moderate to high overall quality; (4) psychiatric comorbidities in patients with the levels comorbidities allowed vs. comorbidities not allowed; (5) psychiatric comorbidities in control participants with the levels comorbidities allowed vs. comorbidities not allowed; (6) patient setting with the levels subgroups recruited from a clinical-setting vs. recruited from a non-clinical setting vs. recruited from both (mixed); (7) medication status with the levels subgroups medicated vs. unmedicated. Separate random-effects models were fitted for each of these variables. Then, a fixed-effects regression including a moderator with the effect estimates of the subgroups was calculated to test whether it significantly moderated SSRT.

Finally, for both meta-regressions and subgroup analyses an omnibus test of moderators was conducted, testing all coefficients excluding the intercept against 0. If the omnibus test reaches significance, it may indicate that some of the heterogeneity could be explained by the predictors included in the model (Viechtbauer, 2010).

### Secondary Outcome Measures of the SST

In addition to the SSRT, the SST identifies other outcome measures that are recommended to be reported (Verbruggen et al., 2019). For the current meta-analysis, we examined stop commission errors, go discrimination errors, go omission errors and go accuracy. All studies that reported these parameters were included in the meta-analyses. When errors were reported in numbers (i.e., means, standard deviations), then the percentage of errors was calculated. The analytical procedures were the same as for the SSRT, except that no meta-regression or subgroup analyses were conducted, due to the smaller number of available studies.

## Results

### Study Selection

**Fig. 1:**
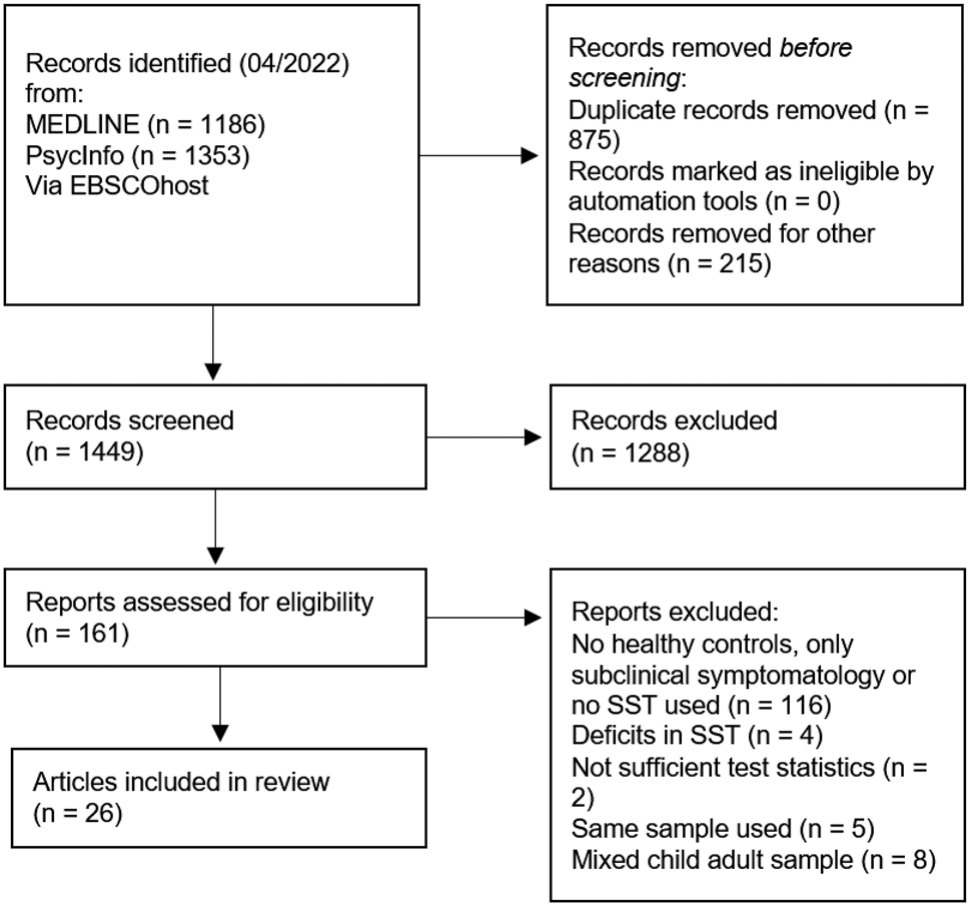
PRISMA flow diagram of study selection (in accordance with Page et al., 2021)

The electronic search resulted in 1186 articles in MEDLINE and 1353 in PsycInfo (Fig. 1). Limiters described in the Methods section excluded 215 of these articles. Search results were exported to EndNote, where EBSCOhost automatically removed 662 duplicates, resulting in 1662 studies. After removing the remaining duplicates using EndNote’s automatic deduplication tool (*n* = 177) and manual inspection (*n* = 36), 1449 articles remained. Screening of titles and abstracts for eligibility resulted in the exclusion of 1288 studies. Full texts of the remaining 161 studies were obtained and checked thoroughly checked for eligibility. Of the 161 studies, 116 were excluded because they did not include a healthy control group (*n* = 1), assessed only subclinical symptomatology (*n* = 2), included ADHD only as comorbid disorder (*n* = 1), or did not use an SST paradigm (*n* = 112). Another 4 studies were excluded because they had substantially modified the SST paradigm and another 8 studies were excluded because their samples included both children and adults. Six studies reported insufficient statistical values for the meta-analysis and authors were contacted. We received data from four studies, which were then included in the final sample. It is important to note that Bekker et al. (2005a, b) and van Dongen-Boomsma et al. (2010) reported identical SSRTs, i.e., for the same experimental session and the same sample of participants. The same accounted for Nigg et al. (2005), Stavro et al. (2007) and Martel et al. (2017) as well as for Linhartová et al. (2020) and Linhartová et al. (2021). For these groups of articles, the reported SSRT value was extracted and counted as a single sample in the meta-analysis. Finally, Szekely et al. (2017) conducted two SST experiments, one implemented for fMRI and one for MEG. Although the samples for these two experiments partially overlapped (63 completed the SST during MEG and fMRI, 85 during fMRI only, and 33 during MEG only), they were treated as single observations in the analysis. Screening of reference lists did not revealed any additional articles. Thus, in total, 26 publications with 27 studies were included in the meta-analysis (1799 participants; ADHD = 883; controls = 916). Sample characteristics for all included studies are shown in Table 1.

**Table 1:** Studies investigating the stop-signal task in adult ADHD

Study	Patient setting	*n*	Males %	Age *M (SD)*	Subtypes *n* (%)	IQ *M (SD)*	Years of education	Medication -/ + (h)	Current CD (*n*)
Adams et al. (2011)	Not enrolled	30 ADHD 27 HC	56.67 48.15	21.1 (1.7) 22.0 (1.7)	*n.a*	103.2 (10.8) V 105.6 (9.8) NV 107.1 (6.9) V 111.2 (8.8) NV	15.1 (1) 15.3 (1.3)	- (24 h)	- -
Aron et al. (2003)	Patient	13 ADHD 13 HC	76.92 61.54	26.2 (6.9) 30.5 (5.0)	3 (23.08) IA 8 (61.54) C 2 (15.38) PR	109 (7.2) V 114 (4.3) V	*n.a*	- (≥ 24 h)	+ -
Bekker et al. (2005a); Bekker et al. (2005b); van Dongen-Boomsma et al. (2010)	Patient	24 ADHD 24 HC	50 50	34.3 (11.68) 34.9 (*n.a.*)	24 (100) C	*n.a*	*n.a*	- (≥ 6 times half-life)	+ -
Bialystok et al. (2017)	Not enrolled	56 (50) ADHD 72 (54) HC	60 29.63	23.55 (4.13) 21.20 (2.74)	*n.a*	100 (12.7) ML NV 103.4 (9.6) ML V 100.9 (13.5) BL NV 100.9 (11.1) BL V 98.1 (12.1) ML NV 102.6 (10.9) ML V 100.7 (14.5) BL NV 95.8 (11.8) BL V	15.5 (2.0) ML 16.1 (2.2) BL 14.9 (1.9) ML 13.9 (1.6) BL	- (24 h)	*n.a*
Boonstra et al. (2010)	Patient	49 ADHD 49 HC	53.06 53.06	38.7 (9.7) 38.1 (9.3)	2 (4.08) HI 47 (95.92) C	100.6 (17.8) 107.71 (16.5)	*n.a*	-	+ -
Chamberlain et al. (2007)	Patient	20 ADHD 20 HC	70 70	31.60 (8.33) 30.90 (7.93)	6 (30) IA 13 (65) C 1 (5) PR	109.9 (9.2) 112.1 (6.2)	*n.a*	- (≥ 12 h)	+ -
Cherkasova et al. (2014)	*n.a*	15 (14) ADHD 18 (12) HC	100 100	29.87 (8.65) 25.44 (6.77)	10 (66.67) IA 5 (33.33) C	107.13 (12.78) 116.83 (16.07)	16.20 (3.63) 17.11 (3.32)	-	- -
Clark et al. (2007)	Patient	20 ADHD 16 HC	65 87.5	28.0 (8.6) 25.1 (5.4)	4 (20) IA 2 (10) HI 10 (50) C 2 (10) PR 2 (10) NOS	108.3 (5.9) 113.3 (3.5)	13.7 (1.7) 14.4 (3.2)	- (24 h)	+ +
Congdon et al. (2014)	*n.a*	25 ADHD 62 HC	56 45	31.24 (10.37) 30.82 (8.97)	*n.a*	*n.a*	14.28 (1.74) 15.10 (1.75)	-	- -
Crunelle et al. (2013)	Patient	17 ADHD 17 HC	100 100	33 (7) 31 (6)	8 (47) IA 9 (53) C	105 (4) 106 (4)	*n.a*	-	+ -
Cubillo et al. (2010)	Not enrolled	10 ADHD 14 HC	100 100	28 (1) 28 (2)	*n.a*	90 (8) 113 (11)	*n.a*	-	+ -
Epstein et al. (2001)	Mixed	25 ADHD 30 HC	40 50	33.6 (*n.a.*) 33.4 (*n.a.*)	14 (56) IA 1 (4) HI 10 (40) C	*n.a*	*n.a*	-	+ +
Hadas et al. (2021)	Not enrolled	52 ADHD 49 HC	80.36 67.31	25.7 (0.5) 26 (0.3)	*n.a*	*n.a*	*n.a*	- (1 week)	- *n.a*
Hamzeloo et al. (2018)	Not enrolled	30 ADHD 30 HC	100	29.38 (6.10)	*n.a*	*n.a*	7.78, (2.48)	*n.a*	+ +
Kamradt et al. (2014)	Mixed	170 ADHD 83 HC	54.7 56.1	23.8 (4.7) 20.1 (2.9)	65 (38.2) IA 10 (5.8) HI 95 (55.8) C	*n.a*	*n.a*	- (24 h to 48 h)	+ +
Lampe et al. (2007)	Patient	22 (16) ADHD 20 (17) HC	63.63 30	29.95 (8.2) 28.7 (6.9)	14 (63.64) IA 1 (4.55) HI 7 (31.82) C	111.00 (11.6) 114.2 (8.5)	*n.a*	- (4 weeks)	- -
Linhartová et al. (2021)	Patient	26 ADHD 26 HC	73 69	23.88 (8.14) 23.69 (7.49)	*n.a*	*n.a*	*n.a*	+	+ -
Marx et al. (2013)	Patient	18 ADHD 20 HC	61 45	27.72 (6.21) 24.75 (3.63)	4 (18.42) IA 1 (2.63) HI 13 (78.95) C	123.33 (16.82) 127.65 (12.33)	*n.a*	- (≥ 72 h)	+ -
Meachon et al. (2021)	*n.a*	9 (8) ADHD 22 (19) HC	33.33 36.36	26.44 (5.83) 23.19 (5.61)	*n.a*	*n.a*	*n.a*	-	+ -
Murphy (2002)	Not enrolled	18 ADHD 18 HC	100 100	27–58 25–59	18 (100) C	110 (9.23) 116 (11.48)	*n.a*	*n.a*	+ +
Nigg et al. (2005); Stavro et al. (2007)	Not enrolled	105 ADHD 90 HC	67.6 35.6	23.70 (4.28) 24.64 (4.77)	26 (24.76) IA 5 (4.76) HI 28 (26.67) C 21 (20) IN 25 (23.08) PR	110.80 (11.59) 113.23 (10.10)	*n.a*	- (≥ 24 h to 48 h)	+ +
Ossmann and Mulligan (2003)	Not enrolled	24 ADHD 24 HC	58.33 58.33	19.21 (1.18) 19.42 (1.06)	*n.a*	116.71 (8.74) 116.33 (10.20)	*n.a*	- (≥ 12 h)	*n.a* *n.a*
Pironti et al. (2014)	Patient	20 ADHD 20 HC	85 65	32.2 (10.31) 32.55 (5.8)	4 (20) IA 16 (80) C	115.26 (6.15) 119.49 (3.27)	*n.a*	- (≥ 24 h)	- -
Roberts et al. (2011)	Not enrolled	30 ADHD 28 HC	56.7 46.43	21.1 (1.7) 22.1 (1.7)	*n.a*	104.9 (10.1) 109.9 (6.9)	15.1 (1.0 15.3 (1.2)	- (≥ 24 h)	+ +
Sebastian et al. (2012)	Patient	20 ADHD 24 HC	55 45.83	33.3 (8.9) 30.3 (8.1)	9 (45) IA 11 (55) C	115.3 (16.7) 115.7 (16.0)	*n.a*	- (≥ 2 months)	+ -
Szekely et al. (2017), fMRI	*n.a*	24 ADHD 84 HC	45.8 57.1	23.34 (3.95) 24.46 (4.09)	*n.a*	*n.a*	*n.a*	- (≥ 24 h)	+ -
Szekely et al. (2017), MEG	*n.a*	25 ADHD 46 HC	52.0 47.8	23.73 (4.18) 23.31 (2.96)	*n.a*	*n.a*	*n.a*	- (≥ 24 h)	+ -

*ADHD* Attention-deficit hyperactivity disorder; *HC* Healthy controls; Patient Setting: ADHD group recruited from in/outpatient setting or non-clinical setting; Years of education: If education status was presented in any other form than years of education, it was not included in this table. Medication: allowed or not allowed during testing (+ and -, respectively). If medication was not allowed, duration of omission prior to testing diplayed in brackets; *CD* Comorbid disorder; *V* Verbal; *NV* Nonverbal; *ML* monolingual; *BL* Bilingual; *IA* Predominantly inattentive subtype; *HI* Predominantly hyperactive-impulsive subtype; *C* Combined subtype; *PR* In partial remission; *NOS* Not otherwise specified; *IN* Inconsistent, met criteria for ADHD–H, ADHD–C, or ADHD–I as children but for a different subtype as adults

Twenty-four out of 27 studies prohibited stimulant medication on the day of testing, two studies did not report this information and one study allowed medication (Linhartová et al., 2021). One study tested the effect of stimulant medication on task performance (Chamberlain et al., 2007) and another study allowed medication during testing (Congdon et al., 2014). To maintain similarity between studies, data from Chamberlain et al. (2007) were extracted for the placebo patient group only, and data from Congdon et al. (2014) were extracted for the unmedicated patient group only. Marx et al. (2013) used an SST paradigm comparing performance with and without reward. For this study only data for the non-reward group were extracted. In some articles, information on the presence of comorbidities or the patient setting was reported ambiguously. For example, Aron et al. (2003) report that healthy controls had “no previous contact with psychiatric services” but it is unclear whether potential comorbidities of healthy controls were screened within the study. Therefore, the coding for these two variables may be biased. On request, Meachon et al. (2021) provided unpublished information on the age and sex distribution in the two groups. Bialystok et al. (2017) provided the mean age and proportion of males for the subset who completed the SST. Demographic variables, information on the IQ and other relevant information on the study population were not available for all studies. A summary is provided in Table 2. A detailed overview of psychiatric comorbidities in patients and in controls is given in Table 3.

**Table 2:** Sample characterization

	ADHD patients	Healthy controls	Total
*N*	883	916	1799
Age (*k* = 26)	27.73 (4.92)	26.98 (4.92)	27.44 (4.72)
Males, % (*k* = 27)	67.19 (19.53)	61.30 (22.66)	64.25 (20.08)
IQ (*k* = 17)	108.41 (7.48)	113.17 (6.08)	110.85 (6.12)
Patient setting, *k*			
Clinical	11	*n.a*	11
Non-clinical	9		9
Mixed	2		2
Comorbidities, *k*			
Allowed	19	7	*n.a*
Not allowed	6	17	
Subtypes, *n* (*k* = 15)			
Primarily inattentive	167	*n.a*	167
Primarily hyperactive	314		314
Combined	22		22
In partial remission	30		30
Inconsistent	21		21
NOS	2		2

*n* Number of participants; *k* Number of studies reporting this information; Males: Percentage of males in the sample; clinical: recruited from a clinical (inpatient/outpatient) setting; non-clinical: recruited from a non-clinical setting (e.g., newspaper, university); mixed: recruited both from a clinical and a non-clinical setting; In partial remission: met at least 6 of 9 DSM-5 criteria in childhood, but only 3 to 5 of 9 criteria in adulthood. Inconsistent: met the diagnostic criteria for a different subtype in childhood than in adulthood; *NOS* Not otherwise specified

**Table 3:** Detailed comorbidity information for patients and healthy controls

	ADHD		Healthy controls	
	*Current*	*Past/Lifetime*	*Current*	*Past/Lifetime*
Adams et al., 2011	None	Depression and/or anxiety (*n* = 5) Alcohol abuse (*n* = 2) Learning disability (*n* = 1)	None	Depression and/or anxiety (*n* = 4) Alcohol abuse (*n* = 1)
Aron et al., 2003	Global Severity Index (GSI) score of the Brief Symptom Inventory^1^: *M* = 1.7 ± .9	*n.a*	Control subjects had no previous contact with psychiatric services	
Bekker et al., 2005a	Depression (*n* = 2) Anxiety disorders (*n* = 8) Substance abuse (*n* = 3)	Depression (*n* = 13) Bipolar disorder (*n* = 1) Tic disorder (*n* = 1) Alcohol dependence (*n* = 1)	Controls reported no psychiatric disorders or developmental disorder in childhood	
Bialystok et al., 2017	*n.a*	*n.a*	*n.a*	*n.a*
Boonstra et al., 2010	Mood disorders (*n* = 13) Anxiety disorders (*n* = 23) Substance abuse (*n* = 7) Substance dependence (*n* = 8) Others (*n* = 6)	Mood disorders (*n* = 18) Anxiety disorders (*n* = 11) Substance abuse (*n* = 14) Substance dependence (*n* = 21) Others (*n* = 6)	Substance dependence (*n* = 2, nicotine)	Mood disorders (*n* = 5) Substance abuse (*n* = 10) Substance dependence (*n* = 6)
Chamberlain et al., 2007	GSI: *M* = 1.28 ± .72	*n.a*	Controls reported no current or history of axis I disorder	
Cherkasova et al., 2014	None	None	None	None
Clark et al., 2007	Exclusion criteria were a current mood, psychotic, or substance-related diagnosis			
Congdon et al., 2014	Participants were excluded for lifetime diagnoses of schizophrenia or other psychotic disorders, bipolar I or II disorder; or current major depressive disorder, suicidality, anxiety disorder (obsessive–compulsive disorder, panic disorder, generalized anxiety disorder, post-traumatic stress disorder), or substance abuse/dependence other than nicotine dependence			
Crunelle et al., 2013	Participants were excluded when currently using any drugs other than alcohol, cannabis, or nicotine		Psychiatric disorders were excluded in healthy controls	
Cubillo et al., 2010	Anxiety disorder (*n* = 1) Mood disorder (*n* = 3) Conduct disorder (*n* = 1) Substance related (*n* = 2)	*n.a*	For healthy controls, exclusion criteria were present or past history of any mental disorder and substance abuse	
Epstein et al., 2001	MDD (*n* = 2) (Hypo)manic episode (*n* = 2) Alcohol abuse/dependence (*n* = 5) Other drug abuse/ dependence (*n* = 1)	*n.a*	Alcohol abuse/ dependence (*n* = 6) Other drug abuse/ dependence (*n* = 1)	*n.a*
Hadas et al., 2021	Neuropsychiatric comorbidities, and use of psychiatric drugs were ruled out		*n.a*	*n.a*
Hamzeloo et al., 2018	Psychiatric comorbidity symptoms for both groups were evaluated (i.e., anxiety, depression, bipolar disorder, PTSD, Antisocial PD and BPD)			
Kamradt et al., 2014	Participants with a history of Tourette’s disorder, schizophrenia or psychosis, or autism spectrum disorder were excluded			
Lampe et al., 2007	None	BPD (*n* = 6) Substance abuse disorder (*n* = 1) Eating disorder (*n* = 4) Anxiety disorder (*n* = 1) PTSD (*n* = 1)	None	None
Linhartová et al., 2021	Comorbid psychotic or affective disorder and addiction lead to exclusion		For healthy controls the absence of psychiatric symptoms was confirmed	
Marx et al., 2013	Depressive disorders (*n* = 2, currently remitted) Adjustment disorder (*n* = 1) Binge-eating (*n* = 2) Personality disorders (*n* = 9, except for BPD)	*n.a*	Within the control group, no psychiatric or personality disorders were observed	
Meachon et al., 2021	ADHD patients reported no history of brain damage or other developmental impairments		The control group additionally reported no history of any psychiatric or other health conditions	
Murphy, 2002	Subjects had to be free of psychosis, major depression, and mania, and were screened for current alcohol and drug abuse or dependence			
	*n.a*	Alcohol abuse/ dependence (*n* = 1) Other drug abuse/ dependence (*n* = 1)	No healthy control met criteria for current alcohol or drug abuse or dependence	
Nigg et al., 2005	Anxiety disorder (*n* = 19) Antisocial PD (*n* = 5)	Alcohol dependence (*n* = 16) Drug dependence (*n* = 11) Substance dependence (*n* = 24) MDD (*n* = 32)	Anxiety disorder (*n* = 11)	Alcohol dependence (*n* = 4) Drug dependence (*n* = 3) Substance dependence (*n* = 7) MDD (*n* = 11)
Ossmann & Mulligan, 2003	*n.a*	*n.a*	*n.a*	*n.a*
Pironti et al., 2014	Participants did not show relevant symptoms of a comorbid disorder reaching clinical significance for a formal DSM-IV Text Revision diagnosis			
Roberts et al., 2011	Depression and/or anxiety (*n* = 5) Alcohol abuse (*n* = 2) Learning disability (*n* = 1)		Depression and/or anxiety (*n* = 4) Bipolar disorder (*n* = 1) Alcohol abuse (*n* = 1)	
Sebastian et al., 2012	Dysthymia (*n* = 4) Social phobia (*n* = 3) Specific phobia (*n* = 2) Anxiety disorder (*n* = 1) Substance abuse (*n* = 1) Dependent PD (*n* = 5) Schizoid PD (*n* = 1)	MDD (*n* = 6) Obsessive compulsive disorder (*n* = 1) Substance abuse (*n* = 4) Substance dependence (*n* = 1) Eating disorder (*n* = 3)	Healthy controls had no lifetime history of axis I or axis II disorders	
Szekely et al., 2017 fMRI	Any comorbid disorder (*n* = 12)		None	
Szekely et al., 2017 MEG	Any comorbid disorder (*n* = 12)		None	

Current: diagnoses that patients and/or healthy controls currently meet the criteria for; Past/Lifetime: diagnoses that patients and/or healthy controls met the criteria for in the past; *BPD* Borderline personality disorder; *PTSD* Post-traumatic stress disorder; *PD* Personality disorder; *MDD* Major depressive disorder

^1^Derogatis, L.R., (1993): Brief Symptom Inventory (BSI): Administrative, Scoring and Procedural Manual. 3rd ed. Minneapolis, MN: National Computer Systems

### SST Validity and Risk of Bias

The validity of the SST was evaluated for all 26 articles using 4 items, resulting in 104 individual ratings. Table 4 provides an overview of the ratings. Nineteen studies (73%) were rated as having low validity, 5 studies (19%) as having moderate and 2 studies (8%) as having high validity. Marginal distributions showed some degree of prevalence bias for all items. This bias was strongest for items 2 and 4. All items showed substantial to perfect interrater agreement (Hallgren, 2012), with no systematic differences between raters (Supplementary Table 1). Overall, most of the studies included in the meta-analysis had a low or moderate quality of the SST.

**Table 4:**
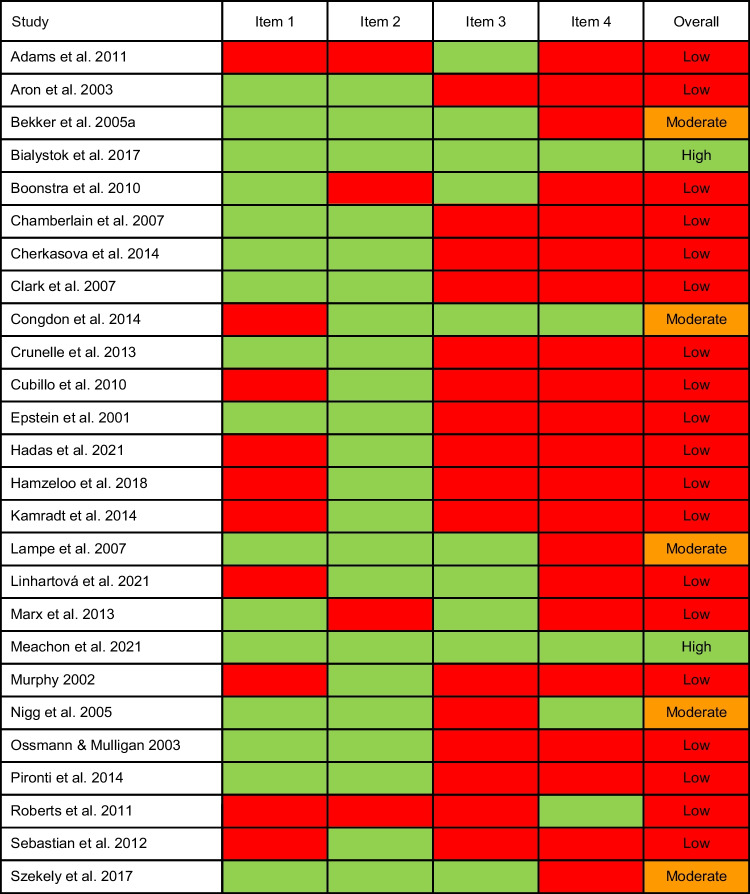
Stop-signal task validity ratings

Item 1: ≥ 50 stop trials in total, stop trials constituting ≤ 25% of all trials; Item 2: staircase algorithm implemented; Item 3: integration method used; Item 4: Cut-Offs applied to ensure valid SSRT estimation; green: fulfilled; red: not fulfilled; Low: 0, 1 or 2 out of 4 items fulfilled; Moderate: 3 out of 4 items fulfilled; High: 4 out of 4 items fulfilled

RoB was evaluated for all 26 articles in 6 domains, resulting in 156 individual ratings. Table 5 provides an overview of the RoB ratings. Overall, most studies had a moderate or high RoB. One article (4%) received a low rating, twelve articles (46%) a moderate rating and 13 articles (50%) a high rating. There was substantial to perfect interrater agreement for all domains (Supplementary Table 2). Two major sources of interrater disagreement were in the reporting of missing data (category 6) and selective outcome reporting (category 4). Some studies did not specifically address whether the entire sample was included in the final SST analysis. However, this could be inferred from the degrees of freedom in the analysis. Therefore, it was decided to use the degrees of freedom to rate this category. In addition, four of the included studies did not report the mean and standard deviation of SST outcomes. Upon request, the authors provided us with these values and the selective data reporting for these four studies was then rated as low RoB.

**Table 5:**
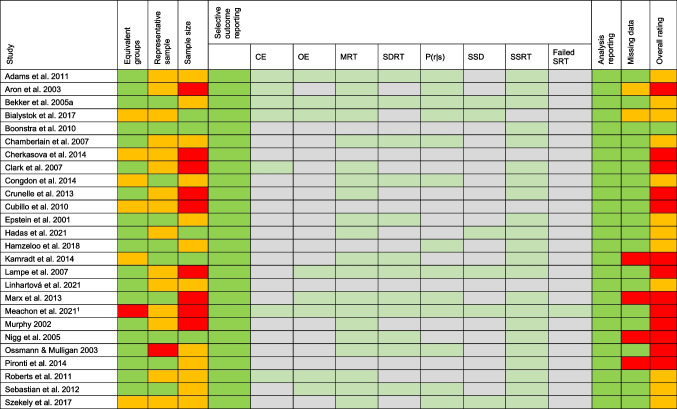
Risk of bias ratings

Ratings were based on the adapted Hombrados and Waddington criteria (Hulsbosch et al., 2021)*; CE* Choice errors; *OE* Omission errors; *MRT* Mean reaction time; *SDRT* Intrasubject variability; *P(r|s)* Probability to respond on a stop trial; *SSD* Stop signal delay; *SSRT* Stop signal reaction time; *Failed SRT* Failed stop reaction time; green: good/low RoB; orange: satisfactory/moderate RoB; red: poor/high RoB. ^1^This study was a pilot study, which might be the reason for small sample sizes

### Meta-Analysis of Stop-Signal Reaction Time

**Fig. 2:**
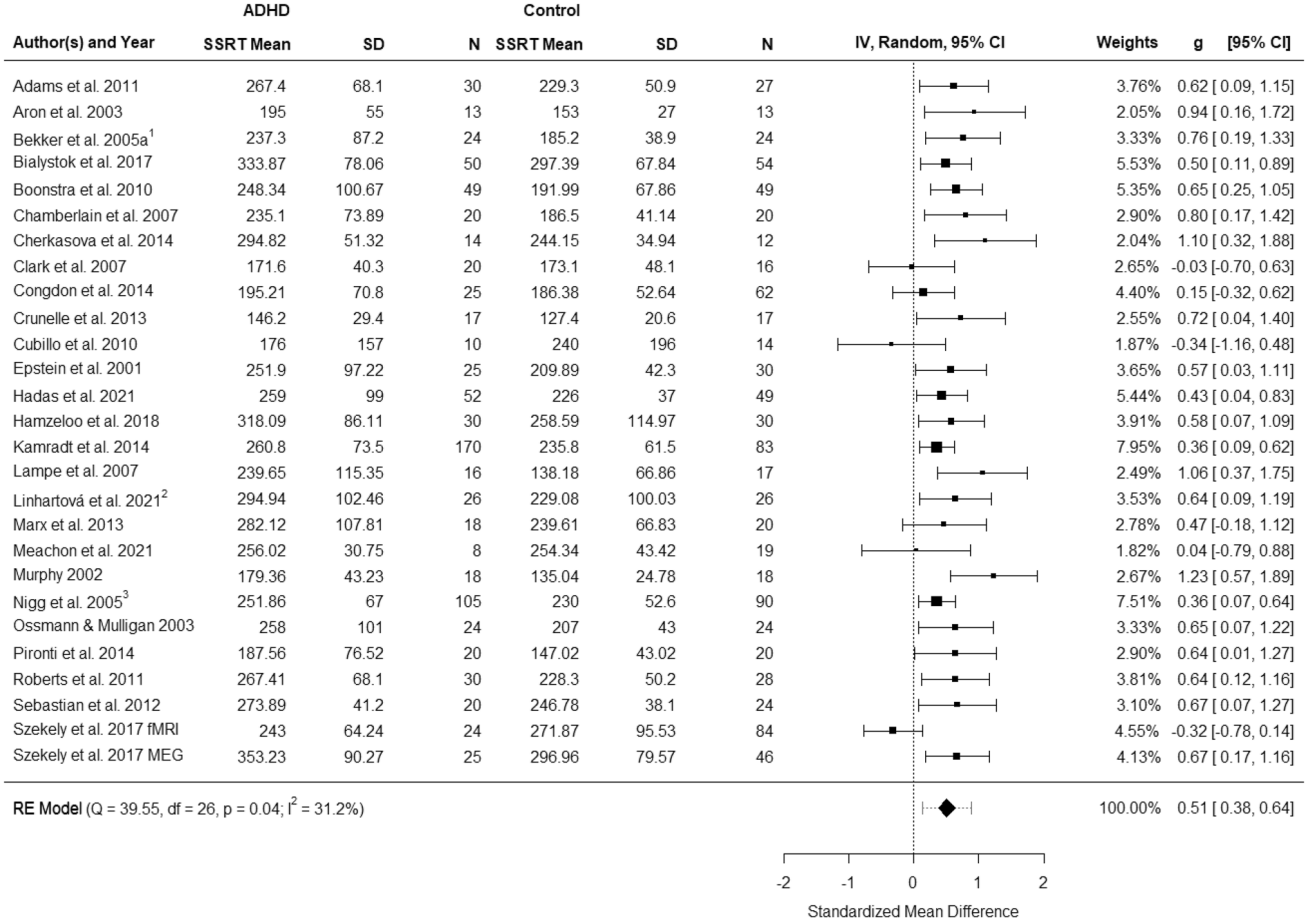
Forest plot showing the observed standardized mean differences (Hedges’ *g*) for SSRT, the random-effects model estimate on the right, and the results of the test for heterogeneity on the left. The dashed line on the overall effect estimate (diamond) represents the prediction interval that is shown due to the present heterogeneity. ^1^Data were extracted from Bekker et al. (2005a, b) and van Dongen-Boomsma et al. (2010); ^2^ Data were extracted from Linhartová et al. (2020) and Linhartová et al. (2021); ^3^Data were extracted from Nigg et al. (2005), Stavro et al. (2007) and Martel et al. (2017)

Figure 2 presents the forest plot of the observed group differences in the SSRT for 27 observations. Across studies, Hedges' $g$ values ranged from -0.341 to 1.230. Results of the random-effects meta-analysis revealed a statistically significant moderate mean effect size estimate of 0.509 (*t*(26) = 7.829, *p* < 0.0001, 95% CI: 0.376–0.644). Adults with ADHD showed moderately higher SSRTs compared to healthy controls. The ${I}^{2}$ statistic demonstrated moderate evidence of heterogeneity across studies (*Q*(26) = 39.546, *p* = 0.043, ${\widehat{\tau }}^{2}=0.030$, ${I}^{2}=31.224\mathrm{\%}$). The heterogeneity reflects in a 95% prediction interval ranging between 0.129 and 0.891.

According to the Cook’s distances, none of the studies was overly influential. However, the study by Szekely et al. (2017) implementing the SST for fMRI had a studentized residual larger than ± 3.113 and is therefore an outlier in the context of this model. Omitting this observation would reduce ${\widehat{\tau }}^{2}$ to 0.000, ${I}^{2}$ to 0.004% and increase $g$ to 0.524 (95% CI 0.416 to 0.631). Linhartová et al. (2021) was the only study that allowed stable stimulant medication during testing and in Chamberlain et al. (2007) patients received a placebo treatment. To explore whether this might have influenced the results, we conducted a sensitivity analysis excluding these two studies. In this analysis $g$ decreased slightly to 0.498 (95% CI 0.354, 0.641), yet heterogeneity remained comparable to the original results with ${\widehat{\tau }}^{2}=0.034$ and ${I}^{2}=34.44\mathrm{\%}$. This indicates that medication does not have a substantial effect on SSRT deficits in adult ADHD. Taken together, the random-effects meta-analysis showed moderate effect sizes $\left(g=0.509\;\mathrm{ to }\;0.524\right)$ with larger SSRTs in patients compared to controls.

### Publication Bias

**Fig. 3:**
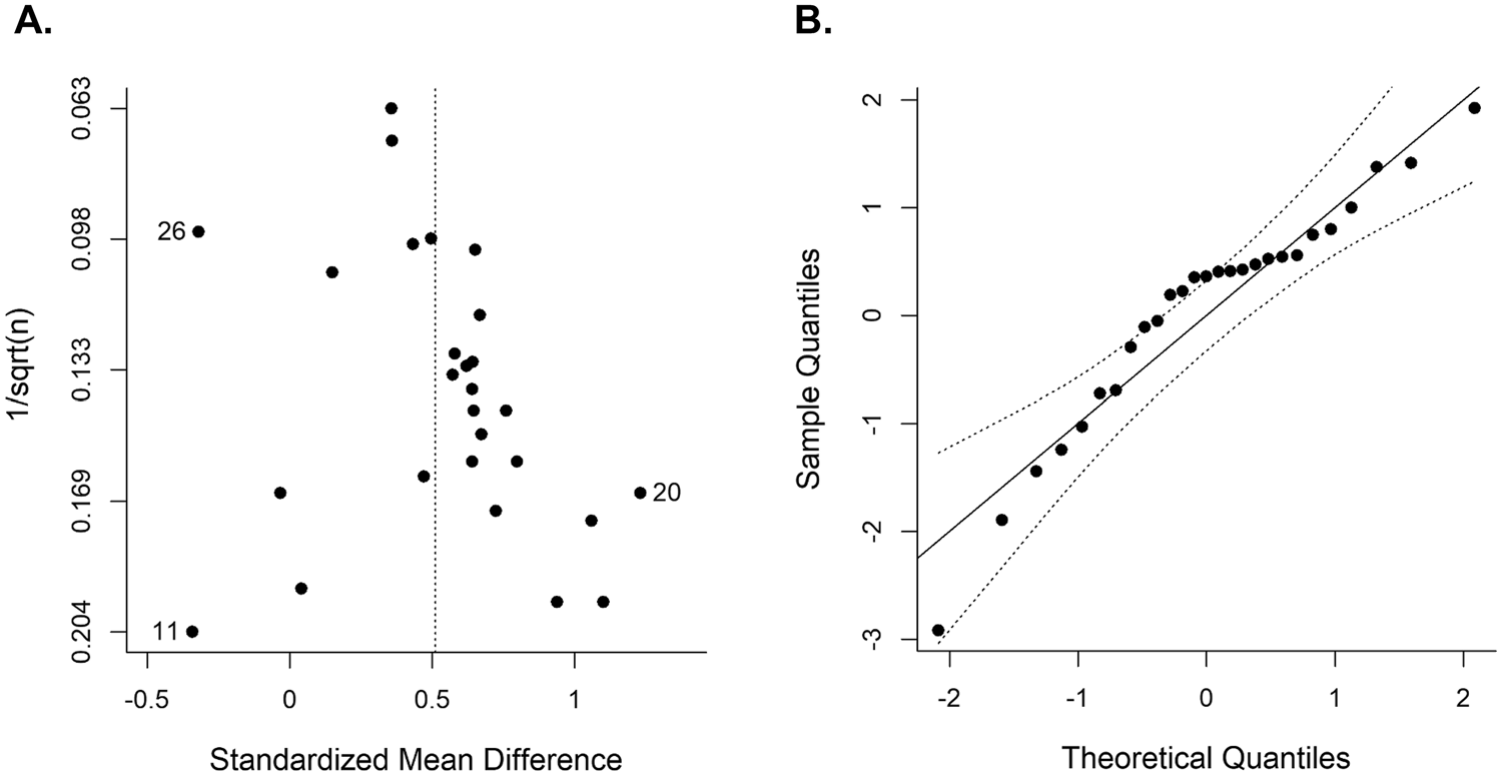
Plots for assessment of publication bias. **A** Funnel plot for SSRT plotting SMDs against the inverse of the square root of the sample size. **B** Normal quantile–quantile plot, plotting the quantiles of a standard normal distribution against the quantiles of the observed distribution. The points should fall on a straight line and inside the 95%-confidence bands. ^11^Cubillo et al. (2010), ^20^Murphy et al. (2002), ^26^Szekely et al. (2017)

Figure 3A depicts a funnel plot of the studies’ SMDs plotted against the inverse of the square root of the sample sizes. Egger’s regression test for funnel plot asymmetry was not significant ($t$(25) = 1.941, $p$ = 0.064). The funnel plot appears to converge close to the mean estimate as the sample size increases. A normal quantile–quantile plot is shown in Fig. 3B. Most of the points in this plot fall inside the 95%-confidence bands. However, there is a slight skewing to the left in the middle of the line, with several points outside the bands. This is an indication that there may be a subtle publication bias.

### Meta-Regression and Subgroup Analysis

Meta-regression analysis was conducted for continuous covariates (age; sex; IQ) and a subgroup analysis for categorical covariates (RoB, SST validity; overall study quality; comorbidities; patient setting; medication status). In this analysis, the data from the fMRI study by Szekely et al. (2017) were an outlier and were therefore excluded from further analysis. In four of the studies (Bialystok et al., 2017; Cherkasova et al., 2014; Lampe et al., 2007; Meachon et al., 2021), only a subset of participants completed the SST. To assess whether this might affect the robustness of the meta-regression results, the analysis was repeated excluding these 4 studies. This did not substantially influence the study outcome (Supplementary Tables 3 and 4). Table 6 provides an overview of the meta-regression analyses. Bialystok et al. (2017) reported demographic and outcome variables separately for monolinguals (ML) and bilinguals (BL) in each group. Values for ML and BL were averaged to obtain only a single value per group for inclusion in the meta-regression analysis. The analysis revealed no significant effects of age, sex or IQ. Table 7 provides an overview of the subgroup analyses. Some studies reported that only some psychiatric comorbidities led to exclusion. These were also coded as “comorbidities allowed”. Data on years of education were sparse and heterogeneous and were therefore not included in the meta-regression. As only one study reported that participants were medicated during testing, medication status was also excluded from the analysis. There were only 5 articles that did not allow for comorbidities in ADHD patients. Therefore, the estimated mean SMD for these studies reported in Table 7 may not be robust. The same accounts for the estimated SMD for the level “mixed” of the setting variable, as only 2 studies reported recruiting ADHD patients from both clinical and non-clinical settings. Interestingly, the differences between the setting subgroups approached significance (*p* = 0.066, Table 7). Therefore, we conducted a follow-up analysis to explore whether there are significant differences between studies with clinical and non-clinical settings only, which was not the case (*p* = 0.171). The analysis of study quality revealed that both RoB assessment and SST validity ratings did not significantly moderate the SSRT. For RoB, the estimated effect was largest for studies with low RoB $\left(g=0.651\right)$ and smallest for studies with high RoB $\left(g=0.531\right)$. However, only one study was classified as having a low RoB, so the result for this category should be interpreted with caution. The group of studies with low SST validity showed the largest average effect size $\left(g=0.556\right)$, whereas the group of studies with high SST validity showed the smallest average effect size $\left(g=0.415\right)$. There were only two studies with high SST validity, which limits the reliability of the result for this category. Similar to RoB, the study quality did not significantly moderate SSRT, with an effect size $g=0.49$ for studies with moderate to high overall quality ratings. Forest plots with subgroups are shown in Supplementary Figs. 1, 2, and 3. In summary, our analysis did not reveal variables that significantly moderated the SSRT deficits in adult ADHD.

**Table 6:** Meta-regression analyses for SSRT

Moderator		*B (SE)*	*t*	*p*	*ci*	*F-*Test	*p*_*F*_
Age, sex (*k* = 25, *n* = 1655)						*F*(3,21) = 0.558	.649
Intercept	0.513 (0.056)		9.182	< .001	0.397, 0.629		
Age	0.085 (0.078)		1.097	.285	-0.077, 0.248		
Sex	-0.003 (0.072)		-0.037	.971	-0.153, 0.148		
Age:Sex	0.054 (0.116)		0.468	.644	-0.187, 0.296		
IQ (*k* = 17, *n* = 937)						*F*(1,15) = 0.345	.566
Intercept	0.601 (0.079)		7.574	< .001	0.432, 0.770		
IQ	0.008 (0.013)		0.587	.566	-0.020, 0.036		

*k* Number of studies for which data was available; *n* Number of participants used for analysis. *B* Regression coefficient. For categorical variables, B is the average estimated effect size for each individual factor level; *SE* Standard error of regression coefficient; *t* T-test for the regression coefficient; *p p*-value for regression coefficient t-test; *CI* Confidence interval; *F-Test* Omnibustest of moderators; *p*_*F*_* p*-value for test of moderators; Sex: Percentage of males in the individual study samples; *IQ* intelligence quotient for ADHD and control group combined

**Table 7:** Subgroup analysis for SSRT

Moderator	*B (SE)*	*z*	*p*	*ci*	*Q*_*M*_*-Test*	*p*_*Q*_
Risk of bias					*Q*_*M*_(2) = 0.260	.878
High (*k* = 13, *n* = 816)	0.531 (0.116)	4.583	< .001	0.304, 0.759		
Moderate (*k* = 12, *n* = 777)	0.023 (0.126)	0.186	0.852	-0.224, 0.271		
Low (*k* = 1, *n* = 98)	0.120 (0.236)	0.508	0.611	-0.342, 0.582		
SST validity					*Q*_*M*_(2) = 0.593	.743
Low (*k* = 19, *n* = 1126)	0.556 (0.062)	8.919	< .001	0.434, 0.678		
Moderate (*k* = 5, *n* = 434)	-0.036 (0.158)	-0.227	0.820	-0.345, 0.273		
High (*k* = 2, *n* = 131)	-0.141 (0.186)	-0.760	0.447	-0.505,0.223		
Overall quality					*Q*_*M*_(2) = 0.173	.917
Low (*k* = 10, *n* = 561)	0.558 (0.140)	3.974	< .001	0.283, 0.833		
Moderate/Low (*k* = 12, *n* = 820)	-0.010 (0.151)	-0.066	0.948	-0.306, 0.286		
Moderate/High (*k* = 4, *n* = 310)	-0.065 (0.189)	-0.343	0.732	-0.435, 0.306		
Psychiatric comorbidities						
In Patients					*Q*_*M*_(1) = 0.132	.716
Allowed (*k* = 18, *n* = 1195)	0.520 (0.065)	7.980	< .001	0.392, 0.648		
Not allowed (*k* = 6, *n* = 344)	0.056 (0.155)	0.363	0.716	-0.247, 0.360		
In Controls					*Q*_*M*_(1) = 1.592	.207
Allowed (*k* = 7, *n* = 693)	0.446 (0.097)	4.584	< .001	0.256, 0.637		
Not allowed (*k* = 16, *n* = 745)	0.157 (0.124)	1.262	0.207	-0.087, 0.400		
Setting					*Q*_*M*_(2) = 5.432	.066
Mixed (*k* = 2, *n* = 308)	0.399 (0.085)	4.698	< .001	0.233, 0.565		
Non-clinical (*k* = 9, *n* = 683)	0.099 (0.123)	0.805	0.421	-0.142, 0.341		
Clinical (*k* = 11, *n* = 489)	0.259 (0.113)	2.288	0.022	0.037, 0.480		

*k* Number of studies for which data was available; *n* Number of participants used for analysis; *B* Regression coefficients (first group is the intercept, for the other groups the coefficients are contrasts); *SE* Standard error of regression coefficient; *z* Wald-type z-test for the regression coefficient; *p p*-value for regression coefficient z-test; *CI* Confidence interval; *Q*_*M*_*-Test* Test for subgroup differences; *p*_*Q*_* p*-value for test for subgroup differences; risk of bias: as assessed by the Hulsbosch Ratings; *SST validity* Stop-signal task validity; overall quality: Risk of bias and SST validity ratings combined; Setting: Setting of recruitment for ADHD group

### Secondary Outcome Measures of the SST

Fifteen studies reported the percentage of stop commissions (Supplementary Fig. 4); 7 studies reported the percentage of choice errors (Supplementary Fig. 5); 9 studies reported omission errors (Supplementary Fig. 6); and 8 studies reported go accuracy (Supplementary Fig. 7). Analysis of the secondary SST outcome measures revealed no significant differences between patients and controls with respect to stop commissions ($g=0.142$, $p=0.064$) and choice errors ($g=0.242$, $p=0.078$). However, ADHD patients made significantly more omission errors ($g=0.418$, $p=0.01$) and had a significantly lower go accuracy ($g=-0.385$, $p<0.008$). A more detailed description of the results is provided in Supplementary Text 3.

## Discussion

In this systematic review and meta-analysis, we integrated the data from 27 studies that examined the stop-signal task in adult ADHD. The analysis revealed inhibitory control deficits, as expressed in prolonged SSRTs, with a moderate effect size $g$ = 0.51. These deficits were not significantly moderated by the study quality, sample characteristics, or clinical parameters. In addition, the analyses of secondary outcome measures revealed greater SST omission errors and reduced go accuracy in patients, although only few studies (*n* < 10) were available for these measures.

### Behavioral Inhibition Deficits in Stop-Signal Response Times

The main finding of our meta-analysis is that patients with adult ADHD reliably show moderate deficits in the SSRT. The magnitude of the deficits is consistent with the results of a previous meta-analysis, which included a much smaller number of studies in adult ADHD (Lipszyc & Schachar, 2010). Our meta-analysis of 27 studies establishes the SSRT as a reliable measure for assessing inhibitory control deficits in adult ADHD. Extending previous work, we evaluated the quality of the SST using the recommendations of a recent consensus paper (Verbruggen et al., 2019) and estimated the risk of bias for each study. The large number of observations allowed us to examine whether study quality, taking into account RoB and the validity of the SST, demographics (age and gender), IQ or clinical parameters (comorbidities and setting) influence SSRT deficits in patients. To this end, we computed meta-regression and subgroup analyses including all studies that reported the respective variables. Surprisingly, none of these variables significantly influenced the magnitude of SSRT deficits in patients. This suggests that the prolonged SSRT in patients can be observed in experimental settings even when the study quality and other parameters are not optimal. The finding that there were no variables which significantly moderated SSRT deficits suggests that deficits in inhibitory control may be a phenotype in adult ADHD.

Another important question is how SSRT deficits relate to clinical symptoms in adult ADHD. In a large-scale study, Kamradt et al. (2014) examined correlations between SSRTs and ratings of current inattentive, hyperactive-impulsive symptoms and executive functions in patients. The study revealed significant moderate relationships between SSRTs and all symptom domains (*r* = 0.23 to 0.30). Using a hierarchical linear regression model that included other neuropsychological paradigms and demographic covariates, the authors found that only the SSRT and the continuous performance test predicted total symptom scores for inattention and hyperactivity-impulsivity. Similarly, Stavro, Nigg and colleagues (Nigg et al., 2005; Stavro et al., 2007) also found moderate (*r* = 0.29) relationships between SSRT deficits and executive functions, as expressed in inattentive-disorganized and hyperactive-impulsive symptoms. Thus, inhibitory control deficits, although frequently reported in empirical studies, are not well reflected in the diagnostic criteria for adult ADHD. For this reason, it could be that these deficits are often neglected during the diagnostic process and therefore also not treated, e.g., in the framework of neurocognitive training.

It is important to note that SSRT deficits are found not only in ADHD but also in other psychiatric disorders such as obsessive–compulsive disorder (OCD), addiction or schizophrenia (Lipszyc & Schachar, 2010; Smith et al., 2014). For example, Lipszyc and Schachar (2010) observed SSRT deficits with effect sizes $g$ = 0.77 and $g$ = 0.69 in OCD and schizophrenia, respectively. However, these analyses included only a few studies (*N* = 4 per group), and an updated evaluation of the SSRT in these groups would be desirable. Nevertheless, given the overlap in inhibitory deficits across disorders, the SST is unlikely to provide diagnostic value in differentiating these disorders. Therefore, we suggest that the SST could be used to quantify inhibitory control deficits in adult ADHD after excluding other psychiatric disorders in which inhibitory control deficits have been reported. In addition to the SST, other paradigms such as the go/no-go task, may be useful in assesssing response inhibition in adult ADHD. For instance, a meta-analysis of the go/no-go task that combined child, adolescent and adult studies, found deficits with a moderate effect size $g$ = 0.49 (Wright et al., 2014), which is comparable with the SSRT deficits in current analysis. In conclusion, the finding of reliable moderate deficits in the SSRT suggests that the SST may become a valuable tool for the neuropsychological assessment of inhibitory control deficits in adult ADHD. To this end, it would be desirable to collect SST data from large samples of participants in order to obtain normative SSRT distributions, considering age, gender and education. An individual’s performance in the SST could then be compared to a normative sample.

### Behavioral Inhibition Deficits in Secondary Measures of the SST

In addition to the SSRT, we computed meta-analyses for stop commission errors, go discrimination errors, go omission errors and go accuracy. These analyses revealed small to moderately greater omission errors $\left(g=0.418\right)$ and reduced go accuracies $\left(g=-0.385\right)$ in patients. However, only a few studies have reported omission errors (*n* = 9) or go accuracy (*n* = 8), and thus, these findings should be interpreted as preliminary evidence.

For omission errors, the study with the largest reported effect size $\left(g=0.73\right)$ was conducted by Roberts et al. (2011). In this study, 30 adult patients with ADHD and 28 control subjects participated in a classical SST paradigm (Logan et al., 1984). Contrary to Roberts et al. (2011), an even negative albeit not significant effect $\left(g=-0.18\right)$ was reported by Bialystok et al. (2017). In their study monolingual and bilingual patients (*n* = 28 monolingual, *n* = 28 bilingual) and controls (*n* = 36 monolingual, *n* = 37 bilingual) participated in a slightly modified version of the SST. Hence, although there was some variance in effect sizes across the studies included in the analysis, on average there were significant small to moderate group differences in omission errors.

A similar variability was also observed for accuracy in go trials, where the largest group differences $\left(g=-0.64\right)$ were reported by Epstein et al. (2001) and the smallest group differences $\left(g=0.24\right)$ were observed by Szekely et al. (Szekely et al., 2017; fMRI study). However, the latter study can be considered as an outlier and a meta-analysis excluding this study resulted in an increased *g* = −0.488. In summary, there is some evidence that, in addition to the SSRT, omission errors and accuracy in go trials during the SST also reflect neurocognitive deficits in adult ADHD. Since the deficits in omission errors and accuracies are restricted to go trials, they suggest an inability to maintain an ongoing response, which is indicative of attentional difficulties. This is in line with previous reports of sustained and focused attention deficits in adult ADHD (Marchetta et al., 2008). Further studies should analyze and report the secondary measures or the SST, which could then be submitted to an updated meta-analysis with a larger number of observations.

### Limitations

This review has some limitations. First, although we used an adapted version of the search syntax proposed by Lipszyc and Schachar (2010) to ensure compatibility with previous reviews, it is possible that the search strategy missed relevant studies by excluding other terms. To ensure that we identified all studies that met our selection criteria, we thoroughly scanned the reference lists of the preselected empirical articles, previous meta-analyses and systematic reviews. Second, the literature search was restricted to peer-reviewed articles written in English or German. This excluded articles that were unpublished or published in a non-commercial form. Therefore, publication bias cannot be excluded. Third, meta-regression analyses based on study-level-averages, such as the mean age of the total study sample carry the risk of an ecological bias. For example, age may be correlated with the outcome within sudies (e.g., Congdon et al., 2014), but not across studies, or vice versa (Higgins & Thompson, 2002). For this reason, the possibility that demographic or clinical variables might influence the results of the SST at the individual study level cannot be completely excluded. Fourth, the validy assessment of the SST revealed that most studies did not use cut-offs in order to identify invalid task behavior. It has been shown that adults with ADHD often failed performance validity measures, i.e., some participants in the studies might have intentionally performed the task incorrectly to mimic cognitive deficits (Marshall et al., 2010, 2016). Therefore, the results of the SSRT meta-analysis results may be biased to some extent. Finally, the quality of most of the studies included in our meta-analysis was not optimal. Therefore, we suggest that future studies should follow recently published best practice recommendations for the design, implementation, analysis and reporting of the SST (Verbruggen et al., 2019) and apply the adapted Hombrados and Waddington criteria to ensure that a representative clinical sample is assessed (Hulsbosch et al., 2021).

## Conclusion

This systematic review and meta-analysis revealed reliable moderate deficits in inhibitory control, as reflected in the SST, in adult ADHD. Our meta-regression and subgroup analyses further demonstrated no significant contribution of demographic and study quality variables on the observed group differences in SSRTs. This suggests that inhibitory control deficits can be considered a phenotype in adult ADHD. Our review and meta-analysis suggest that the SST in conjunction with other neurocognitive tests and clinical questionnaires, could become an important tool for the assessment of inhibitory control deficits in adult ADHD.

### Supplementary Information

Below is the link to the electronic supplementary material.

Supplementary file1 (PDF 341 kb)

## Data Availability

Data sharing is not applicable to this article as no original datasets were generated or analyzed during the current study. A preprint of an earlier version of this work has been made available to the public: https://www.medrxiv.org/content/10.1101/2022.07.09.22277429v1

## References

[CR1] Adams ZW, Roberts WM, Milich R, Fillmore MT (2011). Does response variability predict distractibility among adults with attention-deficit/hyperactivity disorder?. Psychological Assessment.

[CR2] Adler, L. A., Faraone, S. V, Spencer, T. J., Berglund, P., Alperin, S., & Kessler, R. C. (2017). The structure of adult ADHD. *International Journal of Methods in Psychiatric Research*, *26*. 10.1002/mpr.1555 10.1002/mpr.1555 PMC5405726 28211596

[CR3] Aron AR, Dowson JH, Sahakian BJ, Robbins TW (2003). Methylphenidate improves response inhibition in adults with attention-deficit/hyperactivity disorder. Biological Psychiatry.

[CR4] Asherson P, Buitelaar J, Faraone SV, Rohde LA (2016). Adult attention-deficit hyperactivity disorder: Key conceptual issues. The Lancet. Psychiatry.

[CR5] Barkley RA (1997). Behavioral inhibition, sustained attention, and executive functions: Constructing a unifying theory of ADHD. Psychological Bulletin.

[CR6] Bekker EM, Overtoom CC, Kenemans JL, Kooij JJ, De Noord I, Buitelaar JK, Verbaten MN (2005). Stopping and changing in adults with ADHD. Psychological Medicine.

[CR7] Bekker EM, Overtoom CCE, Kooij JJS, Buitelaar JK, Verbaten MN, Kenemans JL (2005). Disentangling deficits in adults with Attention-Deficit/Hyperactivity Disorder. Archives of General Psychiatry.

[CR8] Bialystok E, Hawrylewicz K, Wiseheart M, Toplak M (2017). Interaction of bilingualism and Attention-Deficit/Hyperactivity Disorder in young adults. Bilingualism: Language and Cognition.

[CR9] Boonstra AM, Kooij JJS, Oosterlaan J, Sergeant JA, Buitelaar JK (2010). To act or not to act, that’s the problem: Primarily inhibition difficulties in adult ADHD. Neuropsychology.

[CR10] Byrt T, Bishop J, Carlin JB (1993). Bias, prevalence and kappa. Journal of Clinical Epidemiology.

[CR11] Chamberlain SR, del Campo N, Dowson J, Müller U, Clark L, Robbins TW, Sahakian BJ (2007). atomoxetine improved response inhibition in adults with Attention Deficit/Hyperactivity Disorder. Biological Psychiatry.

[CR12] Cherkasova MV, Faridi N, Casey KF, O’Driscoll GA, Hechtman L, Joober R, Baker GB, Palmer J, Dagher A, Leyton M, Benkelfat C (2014). Amphetamine-induced dopamine release and neurocognitive function in treatment-naive adults with ADHD. Neuropsychopharmacology.

[CR13] Clark L, Blackwell AD, Aron AR, Turner DC, Dowson J, Robbins TW, Sahakian BJ (2007). Association between response inhibition and working memory in adult ADHD: A link to right frontal cortex pathology?. Biological Psychiatry.

[CR14] Cohen J (1968). Weighted kappa: Nominal scale agreement with provision for scaled disagreement or partial credit. Psychological Bulletin.

[CR15] Congdon E, Altshuler LL, Mumford JA, Karlsgodt KH, Sabb FW, Ventura J, McGough JJ, London ED, Cannon TD, Bilder RM, Poldrack RA (2014). Neural activation during response inhibition in adult attention-deficit/hyperactivity disorder: Preliminary findings on the effects of medication and symptom severity. Psychiatry Research: Neuroimaging.

[CR16] Copas J, Chi JQ (2000). Meta-analysis, funnel plots and sensitivity analysis. Biostatistics.

[CR17] Crunelle CL, Veltman DJ, van Emmerik-van Oortmerssen K, Booij J, van den Brink W (2013). Impulsivity in adult ADHD patients with and without cocaine dependence. Drug and Alcohol Dependence.

[CR18] Cubillo A, Halari R, Ecker C, Giampietro V, Taylor E, Rubia K (2010). Reduced activation and inter-regional functional connectivity of fronto-striatal networks in adults with childhood Attention-Deficit Hyperactivity Disorder (ADHD) and persisting symptoms during tasks of motor inhibition and cognitive switching. Journal of Psychiatric Research.

[CR19] Derogatis, L. R. (1993). Brief Symptom Inventory (BSI): Administrative, Scoring and Procedural Manual. 3rd ed. Minneapolis, MN: National Computer Systems.

[CR20] Epstein JN, Johnson DE, Varia IM, Conners CK (2001). Neuropsychological assessment of response inhibition in adults with ADHD. Journal of Clinical and Experimental Neuropsychology.

[CR21] Fisher T, Aharon-Peretz J, Pratt H (2011). Dis-regulation of response inhibition in adult Attention Deficit Hyperactivity Disorder (ADHD): An ERP study. Clinical Neurophysiology: Official Journal of the International Federation of Clinical Neurophysiology.

[CR22] Fried R, DiSalvo M, Kelberman C, Biederman J (2021). Can the CANTAB identify adults with attention-deficit/hyperactivity disorder? A controlled study. Applied Neuropsychology: Adult.

[CR23] Hadas I, Hadar A, Lazarovits A, Daskalakis ZJ, Zangen A (2021). Right prefrontal activation predicts ADHD and its severity: A TMS-EEG study in young adults. Progress in Neuro-Psychopharmacology & Biological Psychiatry.

[CR24] Hallgren KA (2012). Computing inter-rater reliability for observational data: An overview and tutorial. Tutorials in Quantitative Methods for Psychology.

[CR25] Hamzeloo M, Mashhadi A, Fadardi JS, Ghahremanzadeh M (2018). Adult attention deficit/hyperactivity disorder among the prison inmates: An investigation of the executive function differences and comorbidity effects. Australian Journal of Psychology.

[CR26] Higgins JPT, Thompson SG (2002). Quantifying heterogeneity in a meta-analysis. Statistics in Medicine.

[CR27] Hulsbosch A-K, De Meyer H, Beckers T, Danckaerts M, Van Liefferinge D, Tripp G, Van der Oord S (2021). Systematic Review: Attention-Deficit/Hyperactivity Disorder and Instrumental Learning. Journal of the American Academy of Child and Adolescent Psychiatry.

[CR28] Kamradt JM, Ullsperger JM, Nikolas MA (2014). Executive function assessment and adult attention-deficit/hyperactivity disorder: Tasks versus ratings on the Barkley deficits in executive functioning scale. Psychological Assessment.

[CR29] Knapp G, Hartung J (2003). Improved tests for a random effects meta-regression with a single covariate. Statistics in Medicine.

[CR30] Lampe K, Konrad K, Kroener S, Fast K, Kunert HJ, Herpertz SC (2007). Neuropsychological and behavioural disinhibition in adult ADHD compared to borderline personality disorder. Psychological Medicine.

[CR31] Lin L, Aloe AM (2021). Evaluation of various estimators for standardized mean difference in meta-analysis. Statistics in Medicine.

[CR32] Linhartová P, Látalová A, Barteček R, Širůček J, Theiner P, Ejova A, Hlavatá P, Kóša B, Jeřábková B, Bareš M, Kašpárek T (2020). Impulsivity in patients with borderline personality disorder: A comprehensive profile compared with healthy people and patients with ADHD. Psychological Medicine.

[CR33] Linhartová P, Širůček J, Ejova A, Barteček R, Theiner P, Kašpárek T (2021). Dimensions of impulsivity in healthy people, patients with borderline personality disorder, and patients with attention-deficit/hyperactivity disorder. Journal of Attention Disorders.

[CR34] Lipszyc J, Schachar R (2010). Inhibitory control and psychopathology: A meta-analysis of studies using the stop signal task. Journal of the International Neuropsychological Society.

[CR35] Logan GD, Cowan WB, Davis KA (1984). On the ability to inhibit simple and choice reaction time responses: A model and a method. Journal of Experimental Psychology: Human Perception and Performance.

[CR36] Logan GD, Van Zandt T, Verbruggen F, Wagenmakers E-J (2014). On the ability to inhibit thought and action: General and special theories of an act of control. Psychological Review.

[CR37] Marchetta NDJ, Hurks PPM, De Sonneville LMJ, Krabbendam L, Jolles J (2008). Sustained and focused attention deficits in adult ADHD. Journal of Attention Disorders.

[CR38] Marshall P, Schroeder R, O’Brien J, Fischer R, Ries A, Blesi B, Barker J (2010). Effectiveness of symptom validity measures in identifying cognitive and behavioral symptom exaggeration in adult attention deficit hyperactivity disorder. The Clinical Neuropsychologist.

[CR39] Marshall PS, Hoelzle JB, Heyerdahl D, Nelson NW (2016). The impact of failing to identify suspect effort in patients undergoing adult attention-deficit/hyperactivity disorder (ADHD) assessment. Psychological Assessment.

[CR40] Martel MM, Nigg JT, Schimmack U (2017). Psychometrically informed approach to integration of multiple informant ratings in adult ADHD in a community-recruited sample. Assessment.

[CR41] Marx I, Höpcke C, Berger C, Wandschneider R, Herpertz SC (2013). The impact of financial reward contingencies on cognitive function profiles in adult ADHD. PLoS One.

[CR42] Meachon, E. J., Meyer, M., Wilmut, K., Zemp, M., & Alpers, G. W. (2021). Evoked potentials differentiate developmental coordination disorder from Attention-Deficit/Hyperactivity Disorder in a stop-signal task: A pilot study. *Frontiers in Human Neuroscience*, *15*. 10.3389/fnhum.2021.629479 10.3389/fnhum.2021.629479 PMC7990764 33776670

[CR43] Murphy P (2002). Inhibitory control in adults with attention-deficit/hyperactivity disorder. Journal of Attention Disorders.

[CR44] Nigg JT, Butler KM, Huang-Pollock CL, Henderson JM (2002). Inhibitory processes in adults with persistent childhood onset ADHD. Journal of Consulting and Clinical Psychology.

[CR45] Nigg JT, Stavro G, Ettenhofer M, Hambrick DZ, Miller T, Henderson JM (2005). Executive functions and ADHD in adults: Evidence for selective effects on ADHD symptom domains. Journal of Abnormal Psychology.

[CR46] Nikolas MA, Marshall P, Hoelzle JB (2019). The role of neurocognitive tests in the assessment of adult attention-deficit/hyperactivity disorder. Psychological Assessment.

[CR47] Ossmann JM, Mulligan NW (2003). Inhibition and attention deficit hyperactivity disorder in adults. American Journal of Psychology.

[CR48] Page, M. J., McKenzie, J. E., Bossuyt, P. M., Boutron, I., Hoffmann, T. C., Mulrow, C. D., Shamseer, L., Tetzlaff, J. M., Akl, E. A., Brennan, S. E., Chou, R., Glanville, J., Grimshaw, J. M., Hróbjartsson, A., Lalu, M. M., Li, T., Loder, E. W., Mayo-Wilson, E., McDonald, S., McGuinness, L. A., … Moher, D. (2021). The PRISMA 2020 statement: An updated guideline for reporting systematic reviews. *BMJ (Clinical research ed.)*, *372*, n71. 10.1136/bmj.n71 10.1136/bmj.n71 PMC8005924 33782057

[CR49] Pironti VA, Lai M-C, Müller U, Dodds CM, Suckling J, Bullmore ET, Sahakian BJ (2014). Neuroanatomical abnormalities and cognitive impairments are shared by adults with attention-deficit/hyperactivity disorder and their unaffected first-degree relatives. Biological Psychiatry.

[CR50] R Core Team. (2020). R: A language and environment for statistical computing. R Foundation for Statistical Computing, Vienna, Austria. https://www.R-project.org/

[CR51] Raud L, Westerhausen R, Dooley N, Huster RJ (2020). Differences in unity: The go/no-go and stop signal tasks rely on different mechanisms. Neuroimage.

[CR52] Riley RD, Higgins JPT, Deeks JJ (2011). Interpretation of random effects meta-analyses. BMJ.

[CR53] Roberts W, Fillmore MT, Milich R (2011). Linking impulsivity and inhibitory control using manual and oculomotor response inhibition tasks. Acta Pathologica (Amst).

[CR54] Sebastian A, Gerdes B, Feige B, Klöppel S, Lange T, Philipsen A, Tebartz van Elst L, Lieb K, Tüscher O (2012). Neural correlates of interference inhibition, action withholding and action cancelation in adult ADHD. Psychiatry Research: Neuroimaging.

[CR55] Silverstein MJ, Faraone SV, Leon TL, Biederman J, Spencer TJ, Adler LA (2020). The relationship between executive function deficits and DSM-5-defined ADHD symptoms. Journal of Attention Disorders.

[CR56] Slusarek M, Velling S, Bunk D, Eggers C (2001). Motivational effects on inhibitory control in children with ADHD. Journal of the American Academy of Child and Adolescent Psychiatry.

[CR57] Smith JL, Mattick RP, Jamadar SD, Iredale JM (2014). Deficits in behavioural inhibition in substance abuse and addiction: A meta-analysis. Drug and Alcohol Dependence.

[CR58] Song P, Zha M, Yang Q, Zhang Y, Li X, Rudan I (2021). The prevalence of adult attention-deficit hyperactivity disorder: A global systematic review and meta-analysis. Journal of Global Health.

[CR59] Stavro GM, Ettenhofer ML, Nigg JT (2007). Executive functions and adaptive functioning in young adult attention-deficit/hyperactivity disorder. Journal of the International Neuropsychological Society.

[CR60] Szekely E, Sudre GP, Sharp W, Leibenluft E, Shaw P (2017). Defining the neural substrate of the adult outcome of childhood ADHD: A multimodal neuroimaging study of response inhibition. American Journal of Psychiatry.

[CR61] van Dongen-Boomsma M, Lansbergen MM, Bekker EM, Sandra Kooij JJ, van der Molen M, Kenemans JL, Buitelaar JK (2010). Relation between resting EEG to cognitive performance and clinical symptoms in adults with attention-deficit/hyperactivity disorder. Neuroscience Letters.

[CR62] Verbruggen, F., Aron, A. R., Band, G. P., Beste, C., Bissett, P. G., Brockett, A. T., Brown, J. W., Chamberlain, S. R., Chambers, C. D., Colonius, H., Colzato, L. S., Corneil, B. D., Coxon, J. P., Dupuis, A., Eagle, D. M., Garavan, H., Greenhouse, I., Heathcote, A., Huster, R. J., Jahfari, S., Kenemans, J. L., Leunissen, I., Li, C. - S. R., Logan, G. D., Matzke, D., Morein-Zamir, S., Murthy, A., Paré, M., Poldrack, R. A., Ridderinkhof, K. R., Robbins, T. W., Roesch, M., Rubia, K., Schachar, R. J., Schall, J. D., Stock, A. - K., Swann, N. C., Thakkar, K. N., Van Der Molen, M. W., Vermeylen, L., Vink, M., Wessel, J. R., Whelan, R., Zandbelt, B. B., & Boehler, C. N. (2019). A consensus guide to capturing the ability to inhibit actions and impulsive behaviors in the stop-signal task. *Elife*, *8*. 10.7554/elife.46323 10.7554/eLife.46323 PMC6533084 31033438

[CR63] Verbruggen F, Logan GD (2008). Response inhibition in the stop-signal paradigm. Trends in Cognitive Sciences.

[CR64] Verbruggen F, Logan GD (2009). Models of response inhibition in the stop-signal and stop-change paradigms. Neuroscience and Biobehavioral Reviews.

[CR65] Viechtbauer W (2005). Bias and efficiency of meta-analytic variance estimators in the random-effects model. Journal of Educational and Behavioral Statistics.

[CR66] Viechtbauer W (2010). Conducting meta-analyses in R with the metafor package. Journal of Statistical Software.

[CR67] Viechtbauer W, Cheung MW-L (2010). Outlier and influence diagnostics for meta-analysis. Research Synthesis Methods.

[CR68] Wang MC, Bushman BJ (1998). Using the normal quantile plot to explore meta-analytic data sets. Psychological Methods.

[CR69] Wright L, Lipszyc J, Dupuis A, Thayapararajah SW, Schachar R (2014). Response inhibition and psychopathology: A meta-analysis of go/no-go task performance. Journal of Abnormal Psychology.

[CR70] Zwetsloot, P. - P., Van Der Naald, M., Sena, E. S., Howells, D. W., Inthout, J., De Groot, J. A., Chamuleau, S. A., Macleod, M. R., & Wever, K. E. (2017). Standardized mean differences cause funnel plot distortion in publication bias assessments. *Elife*, *6*. 10.7554/elife.24260 10.7554/eLife.24260 PMC5621838 28884685

