# Supplementary material for: Assessing Inhibitory Control Deficits in Adult ADHD: A Systematic Review and Meta-analysis of the Stop-signal Task
Source: Neuropsychol Rev. 2023 Jun 10;34(2):548–67. doi: 10.1007/s11065-023-09592-5 (PMC11166755; doi:10.1007/s11065-023-09592-5)
Supplement: Supplementary file 1 — Supplementary file1 (PDF 341 kb) [file 11065_2023_9592_MOESM1_ESM.pdf]

Supplementary Material

Supplementary text 1

Results of calibration session

In order to increase inter-rater reliability, a calibration session was conducted. This way, the two raters could identify possible sources of disagreement and decide on rules on how to rate ambiguous cases. Moreover, the calibration session revealed a different understanding regarding some of the criteria. Therefore, the following additional rules were applied:

Representative sample. We didn't consider a prior diagnosis or medical records if the diagnosis was not confirmed within the study. A judgment by a consultant psychiatrist which is not further described was not considered an interview.

Sample size. If the sample size of ADHD patients has a different RoB than the sample size of controls, the overall rating for the sample sizes is oriented towards the category with the higher RoB. For example, if the ADHD sample size is n = 20 (therefore rated as having a moderate RoB) and the sample size of controls however is n = 31 (therefore rated as having a low RoB), the overall rating for sample sizes will be moderate RoB.

Analysis reporting. We will not consider whether the method of SSRT estimation was reported, as this aspect is covered by the checklist for SST validity.

Outcome reporting. Mean and SD for SSRT must be reported. Studies who did not report SSRT, but kindly provided us with the data after we contacted them, were also rated as having a low RoB in this category. It had to be apparent that non-significant results were equally reported.

Missing data. If studies do not explicitly report on missing data, however, it is obvious that data from all participants has been used (e.g., if it can be inferred from the degrees of freedom in the analysis), we rated studies as having a low RoB in this domain, even though the original tool requires a clear statement on missing data.

Supplementary Text 2

Assessment of SST validity

This short checklist is based on the consensus guide by Verbruggen et al. (2019) and focuses only on the validity of the assessment and analysis of the SST. Therefore, the four-item checklist refers to the recommendations 3 to 9 in the consensus guide and represent these recommendations.

1. Stop trials

Item**:** Did the task include a sufficient number of stop trials and, furthermore, are stop signals only presented on a minority of trials?

- The task includes 50 or more stop trials AND
- Percentage of stop trials is 25% or less (please see also note).

Note: It is also possible to have a higher percentage of stop signals, additional measures to minimize slowing are required (explicitly instruct participants not to wait and include block-based feedback).

Rating:

- Yes (both conditions fulfilled)
- No (at least one condition is not fulfilled)

2. Tracking procedure

Item: Was the stop-signal delay adapted using a tracking procedure (also called staircase)?

- A tracking procedure was implemented with a sufficient step size (usually 50ms steps are used, 16ms steps are too small).

Rating

- Yes (condition fulfilled)
- No (not fulfilled)

3. Estimation Method for SSRT

Item: Was the integration method used to estimate the SSRT?

- The integration method was used to estimate the SSRT.

Note: There are cases that the integration method is only applied when p(respond|signal) is not about 0.50, otherwise the mean or median method is applied. This case also meets the criterion.

Rating:

- Yes (condition fulfilled)
- No (not fulfilled)

4. Check for invalid estimation of SSRT

Item: SSRT should only be estimated, when the assumptions of the horse race model are not violated. There are different methods to check for non-compliant behaviour, e.g., values for p(respond|signal) were lower than 0.25 or higher than 0.75. Did the researchers inspect behaviour for invalid SSRT estimation for each participant for a potential exclusion?

- A rule for inspection for invalid SSRT estimation was applied for each participant.

Note: There are various methods to identify non-compliant participants like reaction time on unsuccessful stop trials should not be numerically longer than reaction time on go trials. This item is not intended for the exclusion of specific trials within the participants (e.g., exclusion of trials of with reactions shorter than 100ms).

Rating:

- Yes (condition fulfilled)
- No (no condition fulfilled)

Overall Rating of SST validity

- High validity: all four items fulfilled
- Moderate validity: three items fulfilled
- Low validity: two or less items fulfilled

Supplementary Text 3

Secondary outcome measures of the SST

Fifteen studies have reported the percentage of stop commissions. Hedges' g of those studies ranged from -0.234 to 0.660, with 73% of estimates indicating that ADHD patients had a higher stop commission percentage *(Supplementary Figure 4*). The two studies that reported stop commissions but did not use a tracking algorithm displayed larger deviations from 50% than the other studies (i.e., Adams et al. 2011: ADHD *M* = 58.2, HC *M* = 62; Marx et al. 2013: ADHD *M* = 64.1, HC *M* = 57.86). The estimated average Hedges' g based on the random-effects model was *g* = 0.142 (95% CI: -0.009 to 0.293), which did not significantly differ from zero ($t(14)$ = 2.014, p = 0.064). Moreover, there was no significant heterogeneity ($Q(14)$ = 13.519, p = 0.486, $\hat{\tau}^2$ = 0.002, I^2 = 2.757%) with a 95% prediction interval given by -0.039 to 0.324. In addition, there was no indication of outliers as indicated by the studentized residuals (no values larger than ±2.935) and none of the studies could be considered overly influential according to the Cook's distances. Egger's regression test did not indicate funnel plot asymmetry $t(13)$ = 2.037, p = 0.063 *(Supplementary Figure 8).*

Only 7 studies reported the percentage of choice errors, a forest plot is shown in *Supplementary Figure 5.* Hedges' g of those studies ranged from -0.467 to 0.541, with 86% of estimates indicating that ADHD patients made more choice errors. The estimated average based on the random-effects model was g = 0.242 (95% CI: -0.037 to 0.521), which did not significantly differ from zero ($t(6)$ = 2.119, p = 0.078). There was no significant heterogeneity ($Q(6)$ = 7.213, p = 0.302, $\hat{\tau}^2$ = 0.002, I^2 = 2.853%). Clark et al. (2007) had a studentized residual larger than ±2.6901 and may be a potential outlier. Cook's distances, however, revealed no overly influential studies. Leaving the study out leads to an average estimate of *g* = 0.315 (95% CI: 0.125 to 0.505, $t(5)$ = 4.251, p = 0.008), $\hat{\tau}^2$ and I^2 decreases to 0. There were not enough studies to test for funnel plot asymmetry, as at least ten studies are recommended for reliable results (Sterne et al., 2011).

A forest plot with 9 studies that reported omission errors is shown in *Supplementary Figure*

6. The range of Hedge's g was -0.176 to 0.731, with 78% of estimates hinting that most ADHD patients made more omission errors. The estimated average effect based on the random-effects model was g = 0.418 (95% CI: 0.132 to 0.703), which differed significantly from zero ($t(8)$ = 3.373, p = 0.01). The test for heterogeneity reached significance ($Q(8)$ = 15.780, p = 0.046, $\hat{\tau}^2$ = 0.078, I^2 = 48.506%) and the I^2 statistics indicated moderate heterogeneity in the results. For the true outcomes, the 95% prediction interval was -0.285 to 1.120. Bialystok et al. (2017) had a studentized residual larger than ±2.773 and may be a potential outlier as well as potentially over influential according to Cook's distances. Leaving the study out leads to an average estimate of g = 0.524 (95% CI: 0.286 to 0.762, $t(7)$ = 5.206, p = 0.001), $\hat{\tau}^2$ decreases to 0.002 and I^2 decreases to 1.561. However, there were not enough studies to evaluate funnel plot asymmetry.

Finally, eight of the selected studies provided go accuracy. A forest plot of these studies is shown in *Supplementary Figure 7.* Observed Hedges' g ranged from -0.644 to 0.238, with 88% of estimates indicating that go accuracy was lower for ADHD patients. The estimated average was g = -0.385 (95% CI: -0.635 to -0.136), which significantly differed from zero ($t(7)$ = -3.650, p = 0.008). Even though the test for heterogeneity failed to reach significance ($Q(7)$ = 9.786, p = 0.201, $\hat{\tau}^2$ = 0.031, I^2 = 32.09%), I^2 indicated moderate heterogeneity, reflected by a 95% prediction interval between -0.871 and 0.100. The fMRI observation of Szekely et al. (2017) had a studentized residual larger than ±2.734 and may be a potential outlier as well as potentially overinfluential according to Cook's distances. Leaving out this observation increases g to -0.488 (95% CI: -0.608 to -0.368, $t(6)$ = -9.963, p < 0.0001) and decreases both $\hat{\tau}^2$ and I^2 to 0. Again, funnel plot asymmetry could not be evaluated due to the small number of studies.

Supplementary Tables

Supplementary Table 1

	Unadjusted kappa	Adjusted kappa
Item 1	0.920	0.923
Item 2	1	1
Item 3	0.752	0.769
Item 4	0.785	0.846

Supplementary Table 1: Inter-rater reliability for stop signal task validity ratings. Item 1: ≥50 stop trials in total, stop trials constituting ≤25% of all trials; Item 2: staircase algorithm implemented; Item 3: integration method used; Item 4: cut-offs applied to ensure valid SSRT estimation.

Supplementary Table 2

	Weighted kappa
Equivalent groups	0.743
Representative sample	0.792
Sample sizes	0.861
Selective outcome reporting	1
Analysis reporting	1
Missing data	0.667

Supplementary Table 2: Inter-rater reliability for RoB ratings. Domains in accordance with the adapted Hombrados and Waddington criteria (Hulsbosch et al., 2021).

Supplementary Table 3

Moderator		*B (SE)*	*t*	*p*	*ci*	*F*-Test	p_F
Age, Sex (*k* = 21, *n* = 1465)						F(3,17) = 0.885	.469
	Intercept	0.477 (0.054)	8.875	<.001	0.364, 0.591		
	Age	0.122 (0.076)	1.604	.127	-0.039, 0.284		
	Sex	-0.077 (0.083)	-0.922	.370	-0.253, 0.099		
	Age:Sex	0.146 (0.126)	1.161	.262	-0.120, 0.412		
IQ (*k* = 14, *n* = 774)						F(1,12) = 0.098	.759
	Intercept	0.564 (0.088)	6.438	<.001	0.373, 0.755		
	IQ	0.005 (0.016)	0.313	.759	-0.030, 0.040		

Supplementary Table 3: Meta-regression analyses for SSRT. k: number of studies for which data was available; n: number of participants used for analysis; B: unstandardized regression coefficient. For categorical variables, B is the average estimated effect size for each individual factor level; SE: standard error of regression coefficient; t: t-test for the regression coefficient; p: p-value for regression coefficient t-test; ci: confidence interval; F-Test: test of moderator; p_F: p-value for test of moderator; Sex: percentage of males in the individual study samples; IQ: for ADHD and control group combined.

Supplementary Table 4

Moderator	*B (SE)*	*z*	*p*	*ci*	*Q_M-Test*	*p_Q*
Comorbidities						
In Patients					Q_M (1) = 0.679	.410
Allowed (*k* = 17, *n* = 1168)	0.532 (0.066)	8.071	<.001	0.403, 0.662		
Not allowed (*k* = 4, *n* = *285*)	-0.104 (0.127)	-0.824	.410	-0.353, 0.144		
In Controls					Q_M (1) = 1.167	.280
Allowed (*k* = 7, *n* = 693)	0.446 (0.097)	4.584	<.001	0.256, 0.637		
Not allowed (*k* = 13, *n* = 659)	0.133 (0.123)	1.080	.280	-0.108, 0.373		
Setting					Q_M (2) = 4.287	.117
Mixed (*k* = 2, *n* = 308)	0.399 (0.085)	4.698	<.001	0.233, 0.565		
Non-clinical (*k* = 8, *n* = 579)	0.105 (0.136)	0.771	.441	-0.162, 0.371		
Clinical (*k* = 10, *n* = 456)	0.230 (0.112)	2.057	.040	0.011, 0.448		

Supplementary Table 4: Subgroup analysis for SSRT. k: number of studies for which data was available; n: number of participants used for analysis; B: regression coefficients (first group is the intercept, for the other groups the coefficients are contrasts); SE: standard error of regression coefficient; Wald-type z-test for the regression coefficient; p: p-value for regression coefficient z-test; ci: confidence interval; Q_M-Test: test for subgroup differences; p_Q: p-value for test for subgroup differences; Setting: setting of recruitment for ADHD group.

Supplementary Figures

Supplementary Figure 1

Author(s) and Year	ADHD SSRT Mean	SD	N	Control SSRT Mean	SD	N	Weights	g [95% CI]
Aron et al. 2003	195	55	13	153	27	13	7.57%	0.94 [0.16, 1.72]
Cherkasova et al. 2014	294.82	51.32	14	244.15	34.94	12	7.54%	1.10 [0.32, 1.88]
Clark et al. 2007	171.6	40.3	20	173.1	48.1	16	9.29%	-0.03 [-0.70, 0.63]
Crunelle et al. 2013	146.2	29.4	17	127.4	20.6	17	9.04%	0.72 [0.04, 1.41]
Cubillo et al. 2010	176	157	10	240	196	14	7.02%	-0.34 [-1.17, 0.48]
Kamradt et al. 2014	260.8	73.5	170	235.8	61.5	83	19.47%	0.36 [0.09, 0.62]
Marx et al. 2013	282.12	107.81	18	239.61	66.83	20	9.66%	0.47 [-0.18, 1.12]
Murphy 2002	179.36	43.23	18	135.04	24.78	18	9.36%	1.23 [0.57, 1.89]
Ossmann & Mulligan 2003	258	101	24	207	43	24	11.08%	0.65 [0.07, 1.22]
Pironti et al. 2014	187.56	76.52	20	147.02	43.02	20	9.98%	0.64 [0.01, 1.27]
**RE Model** (Q = 16.52, df = 9, p = 0.06; I^2 = 44.2%)							100.00%	0.56 [0.24, 0.88]

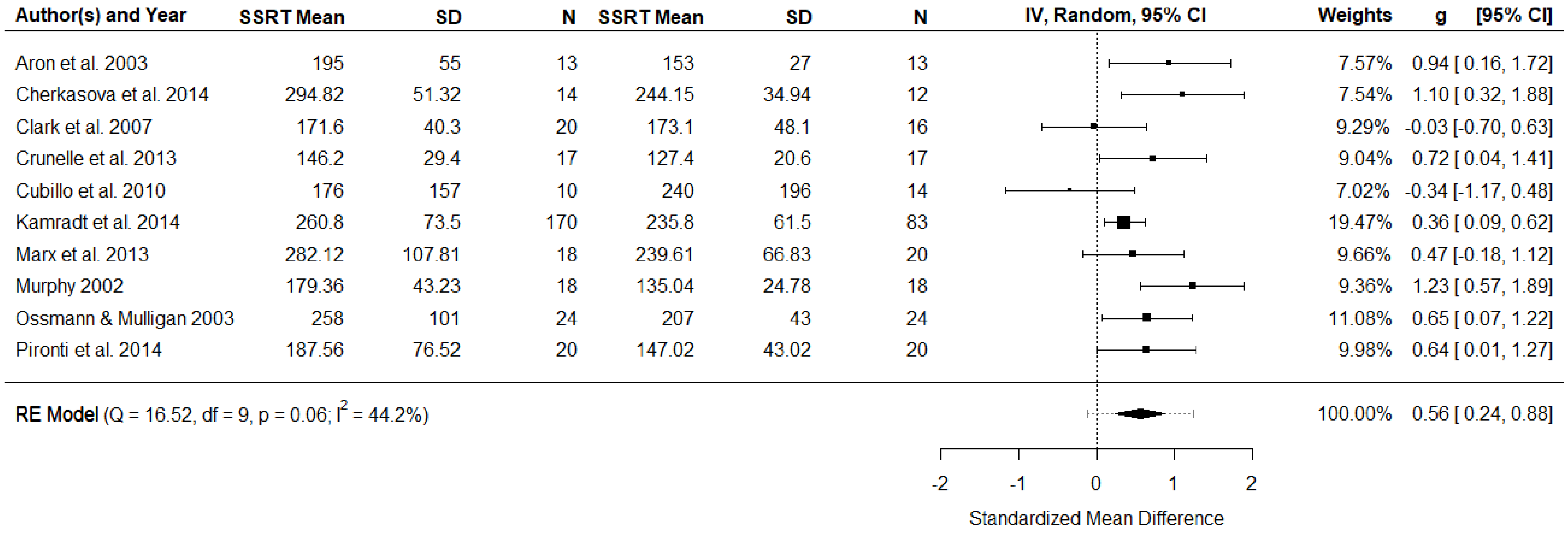

Supplementary Figure 1: SSRT for studies with low quality**.** Forest plot showing the observed standardized mean differences (Hedges' g) for SSRT, the estimates of the random-effects model, and the results for the test of heterogeneity for studies designated as having low quality.

Supplementary Figure 2

	ADHD			Control				
**Author(s) and Year**	**SSRT Mean**	**SD**	**N**	**SSRT Mean**	**SD**	**N**	**Weights**	**g [95% CI]**
Adams et al. 2011	267.4	68.1	30	229.3	50.9	27	6.99%	0.62 [0.09, 1.15]
Boonstra et al. 2010	248.34	100.67	49	191.99	67.86	49	12.05%	0.65 [0.25, 1.05]
Chamberlain et al. 2007	235.1	73.89	20	186.5	41.14	20	4.92%	0.80 [0.17, 1.43]
Epstein et al. 2001	251.9	97.22	25	209.89	42.3	30	6.71%	0.57 [0.03, 1.11]
Hadas et al. 2021	259	99	52	226	37	49	12.41%	0.43 [0.04, 0.83]
Hamzeloo et al. 2018	318.09	86.11	30	258.59	114.97	30	7.38%	0.58 [0.06, 1.09]
Lampe et al. 2007	239.65	115.35	16	138.18	66.86	17	4.05%	1.06 [0.36, 1.75]
Linhartová et al. 2021	294.94	102.46	26	229.08	100.03	26	6.39%	0.64 [0.09, 1.19]
Meachon et al. 2021	256.02	30.75	8	254.34	43.42	19	2.78%	0.04 [-0.80, 0.88]
Nigg et al. 2005	251.86	67	105	230	52.6	90	23.84%	0.36 [0.07, 0.64]
Roberts et al. 2011	267.41	68.1	30	228.3	50.2	28	7.12%	0.64 [0.12, 1.17]
Sebastian et al. 2012	273.89	41.2	20	246.78	38.1	24	5.37%	0.67 [0.07, 1.28]
**RE Model** (Q = 6.84, df = 11, p = 0.81; I^2 = 0.0%)							100.00%	0.55 [0.42, 0.67]

IV, Random, 95% CI

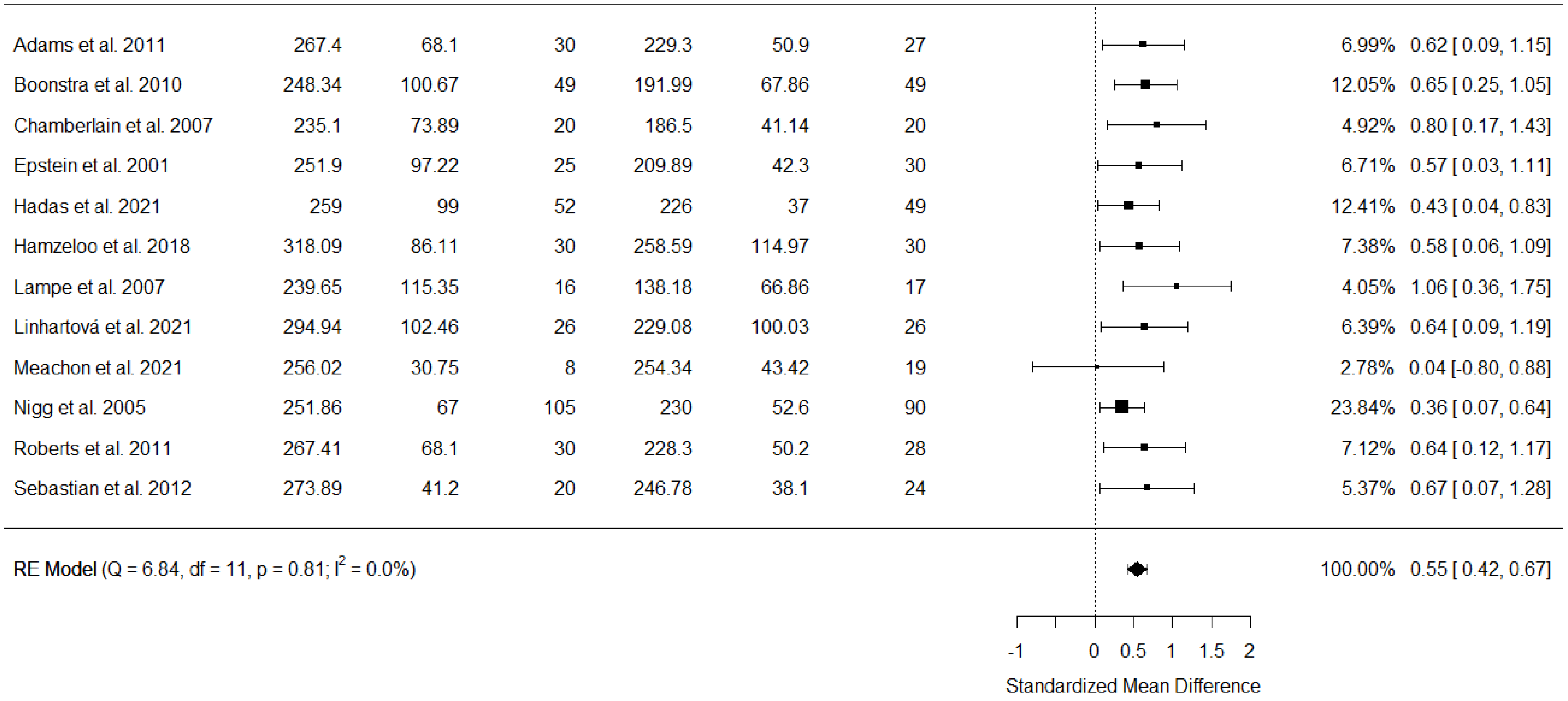

Supplementary Figure 2: SSRT for studies with moderate quality**.** Forest plot showing the observed standardized mean differences (Hedges' g) for SSRT, the estimates of the random-effects model, and the results for the test of heterogeneity for studies designated as having low to moderate quality.

Supplementary Figure 3

	ADHD			Control					
**Author(s) and Year**	**SSRT Mean**	**SD**	**N**	**SSRT Mean**	**SD**	**N**	**IV, Random, 95% CI**	**Weights**	**g [95% CI]**
Bekker et al. 2005a	237.3	87.2	24	185.2	38.9	24		16.87%	0.76 [0.18, 1.33]
Bialystok et al. 2017	333.87	78.06	50	297.39	67.84	54		35.57%	0.50 [0.11, 0.89]
Congdon et al. 2014	195.21	70.8	25	186.38	52.64	62		24.91%	0.15 [-0.32, 0.62]
Szekely et al. 2017 MEG	353.23	90.27	25	296.96	79.57	46		22.65%	0.67 [0.17, 1.16]
**RE Model** (Q = 3.34, df = 3, p = 0.34; I^2 = 3.2%)								100.00%	0.49 [0.09, 0.90]

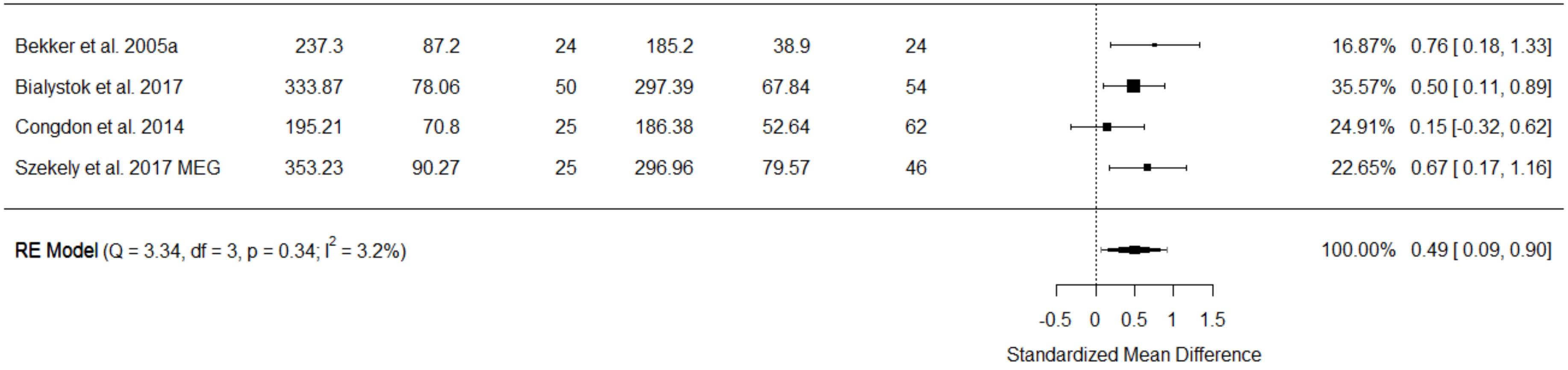

Supplementary Figure 3: SSRT for studies with high quality**.** Forest plot showing the observed standardized mean differences (Hedges' g) for SSRT, the estimates of the random-effects model and the results for the test of heterogeneity for studies designated as having moderate to high quality.

Supplementary Figure 4

	ADHD			Control					
**Author(s) and Year**	**SC Mean**	**SD**	**N**	**SC Mean**	**SD**	**N**	**Weights**	**g**	**[95% CI]**
Adams et al. 2011	58.2	22.9	30	62	20.8	27	7.30%	-0.17	[-0.69, 0.35]
Bekker et al. 2005a	57.24	10.58	24	53.58	6.48	24	6.19%	0.41	[-0.16, 0.98]
Bialystok et al. 2017	46.5	11	50	45	11	54	13.01%	0.14	[-0.25, 0.52]
Chamberlain et al. 2007	51	17	20	45	14	20	5.18%	0.38	[-0.24, 1.00]
Congdon et al. 2014	50.62	9.46	25	50.55	5.7	62	9.09%	0.01	[-0.45, 0.47]
Cubillo et al. 2010	47	4	10	46	5	14	3.05%	0.21	[-0.60, 1.02]
Hamzeloo et al. 2018	47.67	7.87	30	49.73	14.35	30	7.69%	-0.18	[-0.68, 0.33]
Lampe et al. 2007	50.06	7.13	16	48.12	2.55	17	4.29%	0.36	[-0.33, 1.04]
Linhartová et al. 2021	49.28	11.91	26	42.61	7.49	26	6.70%	0.66	[0.12, 1.20]
Marx et al. 2013	64.1	17.53	18	57.86	23.89	20	4.92%	0.29	[-0.35, 0.93]
Meachon et al. 2021	49	2	8	48	3	19	2.95%	0.35	[-0.48, 1.18]
Ossmann & Mulligan 2003	51	11	24	47	3	24	6.19%	0.49	[-0.08, 1.05]
Sebastian et al. 2012	48.7	6.7	20	47.58	6.3	24	5.64%	0.17	[-0.42, 0.76]
Szekely et al. 2017 fMRI	49.06	7.37	24	49.63	8.65	84	9.51%	-0.07	[-0.52, 0.39]
Szekely et al. 2017 MEG	50.98	6.44	25	52.61	7.12	46	8.29%	-0.23	[-0.72, 0.25]
**RE Model** (Q = 13.52, df = 14, p = 0.49; I^2 = 2.8%)							100.00%	0.14	[-0.01, 0.29]

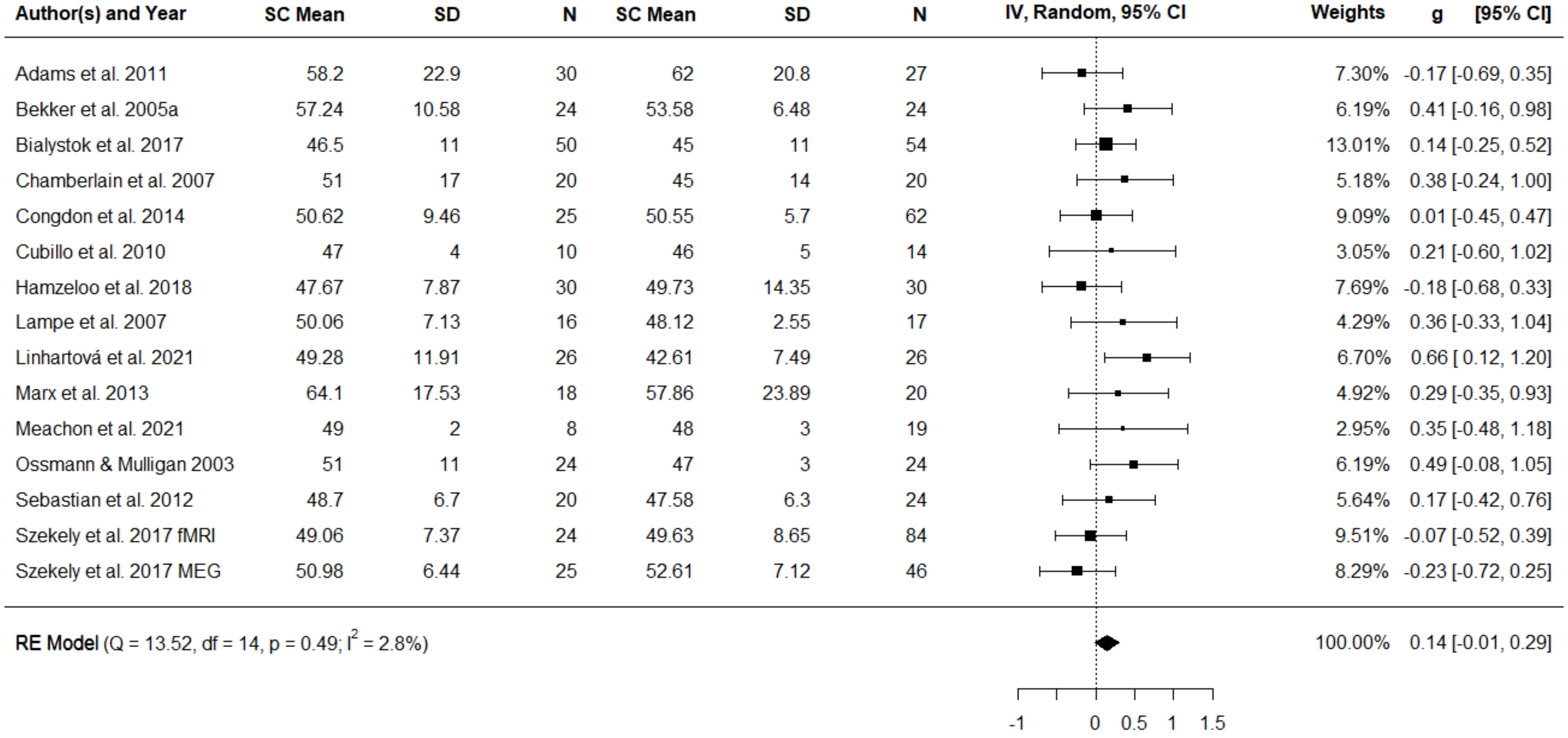

Supplementary Figure 4: Forest plot showing the observed standardized mean differences (Hedges' g) for stop commissions (SC, %), the estimate of the random-effects model and the results for the test of heterogeneity.

Supplementary Figure 5

Author(s) and Year	ADHD CE Mean	SD	N	Control CE Mean	SD	N	Weights	g [95% CI]
Adams et al. 2011	1.04	1.88	30	0.54	0.83	27	15.15%	0.33 [-0.19, 0.86]
Bekker et al. 2005a	3.43	2.7	24	2.12	2.16	24	12.85%	0.53 [-0.04, 1.09]
Bialystok et al. 2017	4.7	5.25	50	4.15	4.7	54	26.98%	0.11 [-0.28, 0.50]
Clark et al. 2007	1.25	1.17	20	2.5	3.71	16	9.59%	-0.47 [-1.13, 0.19]
Linhartová et al. 2021	6.43	13.68	26	1.07	1.89	26	13.89%	0.54 [-0.00, 1.09]
Meachon et al. 2021	4	4	8	3	2	19	6.12%	0.36 [-0.47, 1.19]
Roberts et al. 2011	1.04	1.88	30	0.63	0.94	28	15.43%	0.27 [-0.25, 0.79]
**RE Model** (Q = 7.21, df = 6, p = 0.30; I^2 = 2.9%)							100.00%	0.24 [-0.04, 0.52]

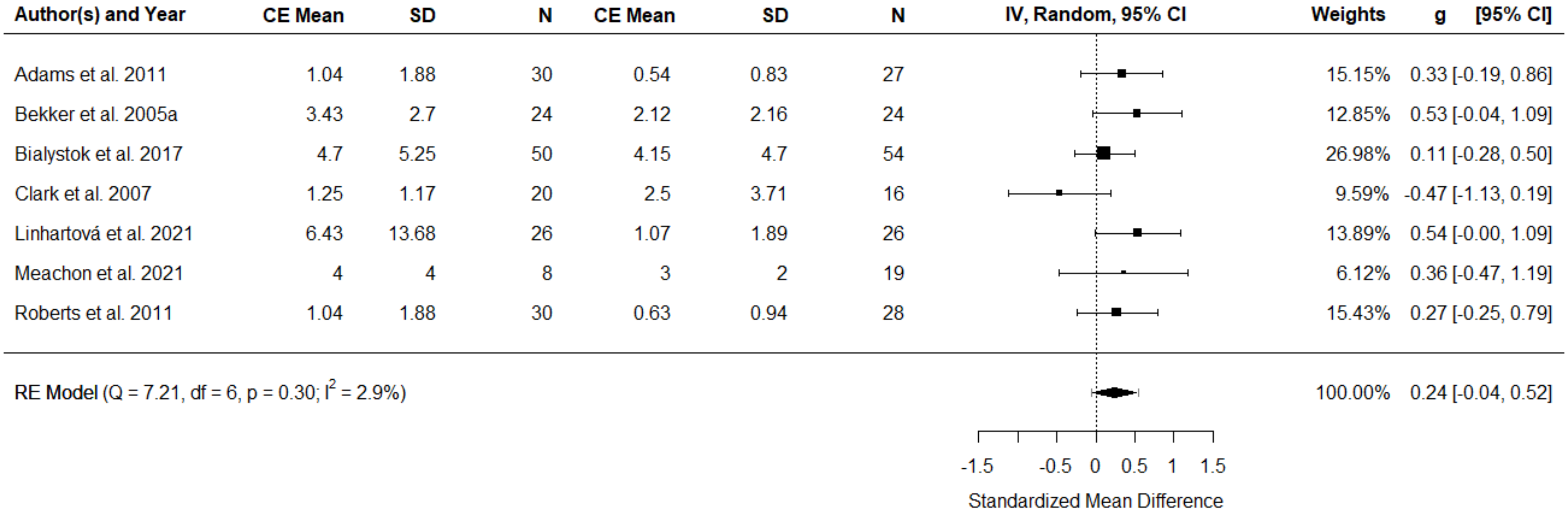

Supplementary Figure 5: Forest plot showing the observed standardized mean differences (Hedges' g) for choice errors (CE) in go trials (%), the estimate of the random-effects model and the results for the test of heterogeneity.

Supplementary Figure 6

	ADHD			Control					
**Author(s) and Year**	**OE Mean**	**SD**	**N**	**OE Mean**	**SD**	**N**	**IV, Random, 95% CI**	**Weights**	**g [95% CI]**
Adams et al. 2011	3.23	4.58	30	0.77	1.15	27		12.25%	0.71 [0.19, 1.23]
Bekker et al. 2005a	2.63	3.05	24	1.22	1.18	24		11.25%	0.60 [0.03, 1.17]
Bialystok et al. 2017	1.1	1.85	50	1.55	3.05	54		15.65%	-0.18 [-0.56, 0.21]
Lampe et al. 2007	1.67	1.78	16	0.64	1.02	17		9.09%	0.70 [0.01, 1.39]
Linhartová et al. 2021	7.88	10.38	26	8.51	15.67	26		11.73%	-0.05 [-0.59, 0.50]
Marx et al. 2013	4.58	6.03	18	1.89	3.09	20		9.88%	0.56 [-0.08, 1.20]
Meachon et al. 2021	4	4	8	2	2	19		7.09%	0.71 [-0.12, 1.55]
Roberts et al. 2011	3.23	4.58	30	0.73	1.04	28		12.36%	0.73 [0.21, 1.25]
Sebastian et al. 2012	2.53	4.3	20	1.23	3.2	24		10.70%	0.34 [-0.26, 0.94]
**RE Model** (Q = 15.78, df = 8, p = 0.05; I^2 = 48.5%)								100.00%	0.42 [0.13, 0.70]

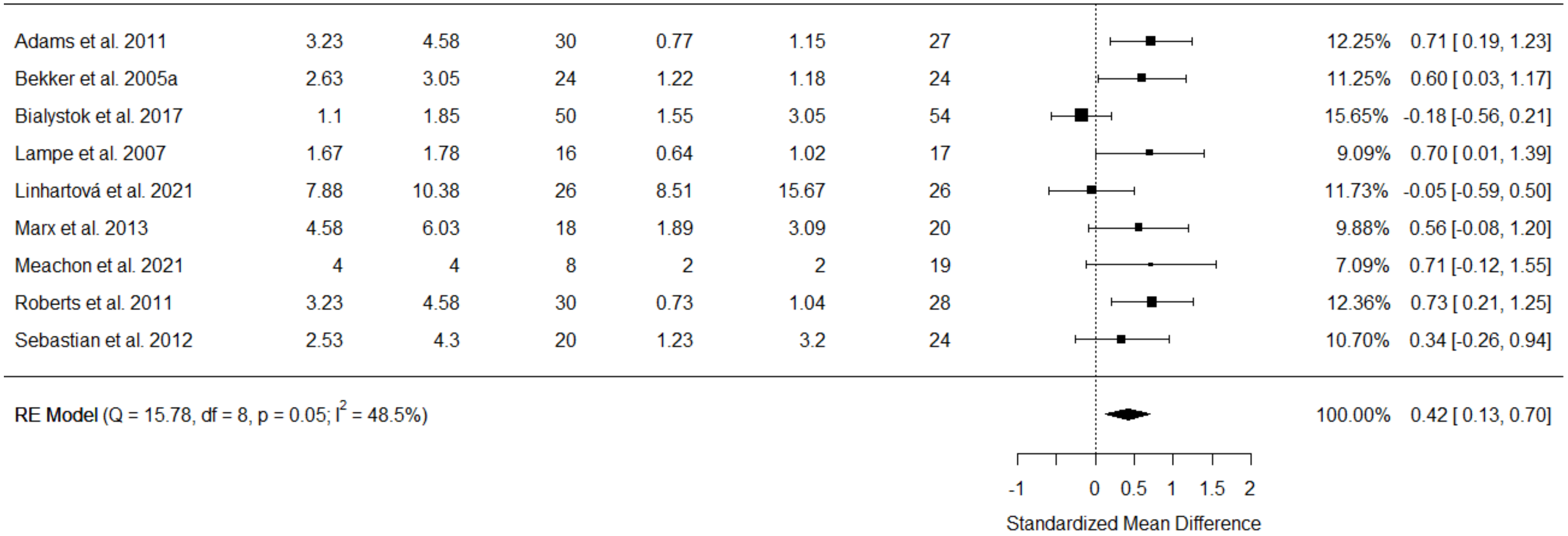

Supplementary Figure 6: Forest plot showing the observed standardized mean differences (Hedges' g) for omission errors (OE) in go trials (%), the estimate of the random-effects model and the results for the test of heterogeneity.

Supplementary Figure 7

Author(s) and Year	ADHD ACC Mean	N	SD	Control ACC Mean	SD	N	IV, Random, 95% CI	Weights	g [95% CI]
Congdon et al. 2014	97.79	6.38	25	99.18	1.76	62		13.92%	-0.37 [-0.84, 0.10]
Crunelle et al. 2013	97.6	2.7	17	98.9	1.8	17		8.13%	-0.55 [-1.23, 0.12]
Epstein et al. 2001	96.39	3.89	25	98.28	1.67	30		11.60%	-0.64 [-1.18, -0.11]
Hadas et al. 2021	89	14	52	94	4	49		17.16%	-0.48 [-0.87, -0.08]
Linhartová et al. 2021	85.7	19.9	26	90.42	15.28	26		11.21%	-0.26 [-0.81, 0.29]
Ossmann & Mulligan 2003	96	4	24	98	3	24		10.58%	-0.56 [-1.13, 0.01]
Szekely et al. 2017 fMRI	91.78	8.36	24	89.43	10.17	84		14.36%	0.24 [-0.22, 0.69]
Szekely et al. 2017 MEG	80.67	8.56	25	86.65	10.5	46		13.06%	-0.60 [-1.09, -0.11]
**RE Model** (Q = 9.79, df = 7, p = 0.20; I^2 = 32.1%)								100.00%	-0.39 [-0.63, -0.14]

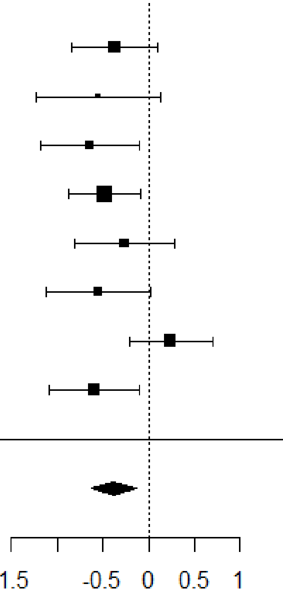

Supplementary Figure 7: Forest plot showing the observed standardized mean differences (Hedges' g) for accuracy (ACC) in go trials (%), the estimate of the random-effects model, and the results for the test of heterogeneity.

Supplementary Figure 8

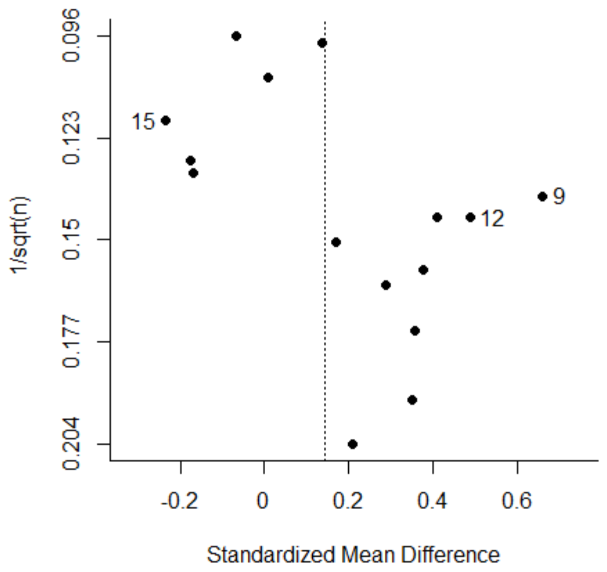

Supplementary Figure 8: Funnel plot for stop commissions plotting SMDs against the inverse of the square root of the sample size. [9]Linhartová et al. (2021); [12]Ossman & Mulligan (2003); [15]Szekely et al. (2017) MEG.
